# Advanced security framework for 5G-SDN attack detection and mitigation using Affinity Cohesive Clustering and Improved Chaotic Walrus-Optimized Convolutional LSTM with Deep Dual Info-Varcoder

**DOI:** 10.3389/frai.2026.1832131

**Published:** 2026-09-03

**Authors:** Shameli R., Sujatha Rajkumar

**Affiliations:** School of Electronics Engineering, Vellore Institute of Technology, Vellore, Tamil Nadu, India

**Keywords:** 5G network security, Affinity Cohesive Clustering, Chaotic Walrus Optimization, Dense Convolutional Long Short-Term Memory, Dual Info Varcoder, Software-Defined Networking

## Abstract

**Introduction:**

The integration of 5G and Software-Defined Networking (SDN) has introduced new security challenges due to limited processing capabilities, inefficient resource allocation, frequent user mobility, and the rapid growth of Internet of Things (IoT) devices. These dynamic network conditions increase the likelihood of malicious activities, highlighting the need for robust and intelligent intrusion detection techniques.

**Methods:**

This research introduces a hybrid intrusion detection framework, termed APCHC-DDST-IV-CLSTM-ICWO, which integrates Affinity Propagated Cohesive Hierarchical Clustering, Deep Dual Stream SpaTemp Info-Varcoder, Dense Convolutional Long Short-Term Memory (CLSTM), and Improved Chaotic Walrus Optimization. The clustering module identifies attack patterns, while the dual-stream Info-Varcoder extracts representative spatial and temporal features. The Dense CLSTM captures sequential dependencies in network traffic to improve attack classification, and the Improved Chaotic Walrus Optimisation algorithm optimises the model parameters to enhance predictive performance. The proposed approach was validated using a 5G-SDN dataset generated with the MATLAB SDN Toolbox, with network traffic and attack scenarios simulated through the NS-3 network simulator.

**Results:**

The model showed a superior classification performance with a high average accuracy of 0.985±0.002, precision of 0.984±0.003, and F1 score of 0.985±0.003. The model’s low log-loss of 0.411 allows for the accurate separation of attack categories, including unknown attacks. This method improves the security and performance of 5G-SDN by increasing the throughput to 3218 samples/second and the detection time to 1.74 seconds.

**Discussion:**

The results of the experiment demonstrate that the proposed APCHC-DDST-IV-CLSTM-ICWO framework offers a feasible method to secure 5G-SDN networks. The system provides high detection accuracy, effective processing, and reliable identification of emerging cyber threats by combining hierarchical clustering, deep feature learning, sequence modeling, and optimization algorithms. These findings indicate that it is appropriate for improving the operational effectiveness and security of next-generation 5G-SDN configurations.

## 1 Introduction

Incorporating 5G with SDN poses many security problems that include security vulnerabilities such as network slicing attacks and increased cyber threat surface area. This is because of the increased number of data transmissions and rapid growth in interconnected devices. Therefore, there is a need to safeguard privacy and data integrity. This becomes more important in the context of smart city infrastructure since connectivity is crucial in achieving efficient and safe operation. To overcome such security vulnerabilities, it is necessary to implement proper security measures to provide secure and resilient 5G-SDN architecture (Aguayo et al., 2023; Bakar et al., 2024; Haider et al., 2020; AbdulRaheem et al., 2024; Su et al., 2023; Paolucci et al., 2021). But one such attack in a 5G-based SDN network is the flow table overloading attack, which floods the network controller with a high volume of flow table entries, exceeding its capacity and causing a denial-of-service (DoS) attack on legitimate traffic. In this way, the SDN controller resources are overloaded by attackers. Therefore, key utilities like emergency systems, transportation systems, and health monitoring suffer from major disruptions, due to which there is great danger to the safety of people in addition to the smart city infrastructure. There are various issues associated with algorithms that are intended to deal with the issue of overload attacks on flow tables due to the highly evolving and advanced nature of attacks, which makes it difficult for the algorithms to be able to cope with them (AlAblani and Arafah, 2023; Tankovic et al., 2025; Cui et al., 2020; Abdulqadder et al., 2023; Aanandaram et al., 2024).

However, some of the existing approaches to attack mitigation in network security can bring an additional overhead or delay, which is undesirable when dealing with 5G-type networks that demand minimal delays. Moreover, the problem increases even further in the case of dynamic environments such as SDN-based networks, since it is rather challenging to make a distinction between legitimate traffic and malicious traffic in such networks. The inability to distinguish between those two leads to false positives/negatives and, therefore, decreases the efficiency of attack detection approaches. Finally, there is another recently emerged threat in 5G-type networks called the Cross-cascaded attack (or crosstalk attack). It refers to the ability of attackers to use the same spectrum for communication in order to capture the legitimate users’ traffic, thus putting the confidentiality of users at risk. In the case of 5G-SDN networks, crosstalk attacks affect the communication channels of SDN controllers. Besides, there is also another threat that should be mentioned, a Fraggle attack. It is a kind of denial-of-service (DoS) attack involving the sending of many UDP echo requests to network broadcast addresses. It utilizes the high bandwidth of 5G networks. The impact of these kinds of attacks on 5G-SDN is severe. The integration of 5G and SDN technologies results in many improvements, but at the same time creates opportunities for potential attacks that exploit the flexibility and scalability of such networks. In turn, the crosstalk and Fraggle attacks demonstrate the necessity to develop advanced and adaptive solutions to protect against these threats. Through the implementation of detection algorithms and proactive defense measures, 5G-SDN networks eliminate these weaknesses. The sources provided discuss the ways in which 5G-SDN networks address crosstalk and Fraggle attacks (Fernández-Carrasco et al., 2022; Whitworth et al., 2023; Ampririt et al., 2023; Qureshi, 2024; Bhardwaj et al., 2022).

**Figure 1:**
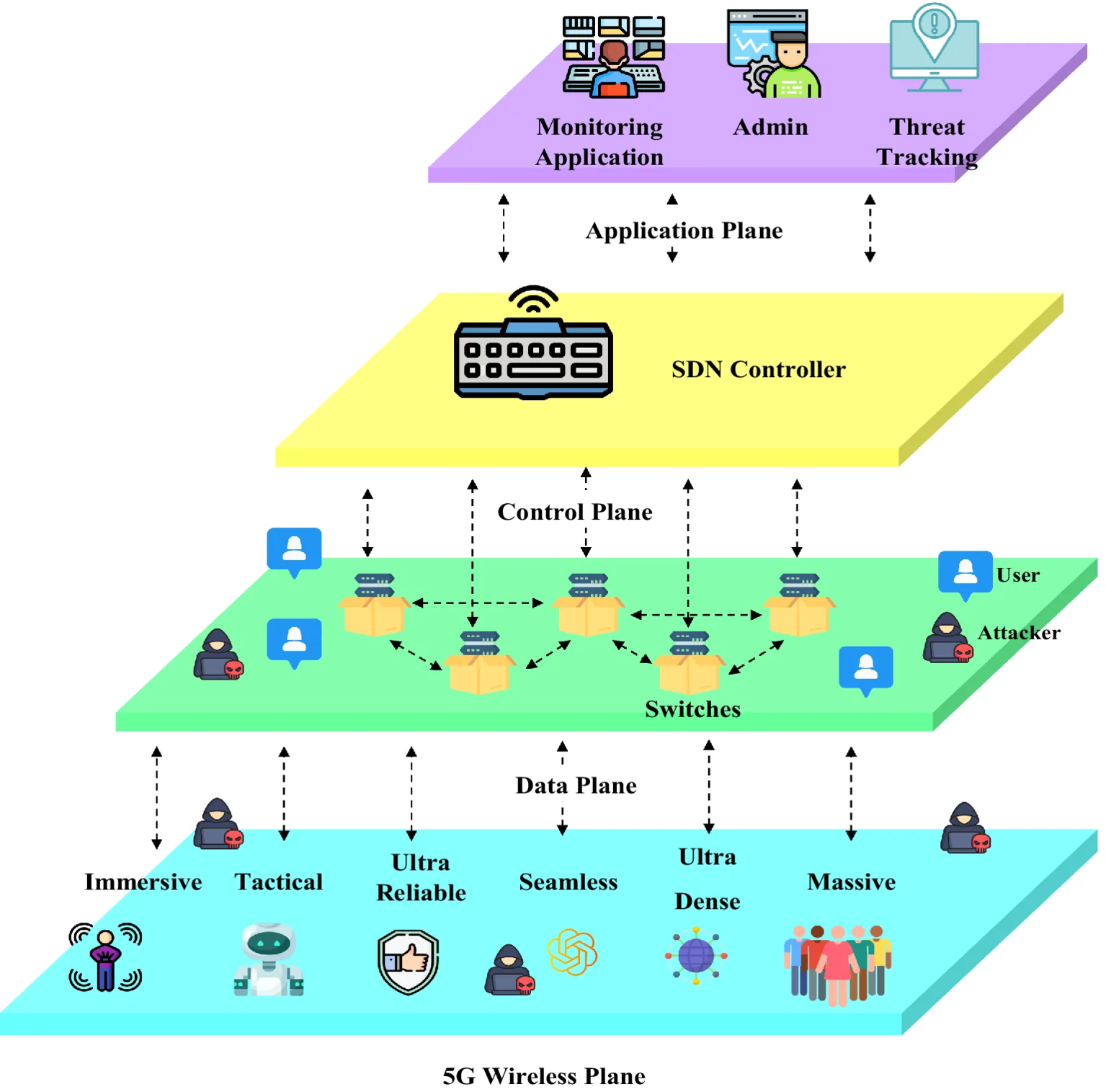
General system architecture of a 5G SDN network.

Some of the problems that the current algorithms for the detection and mitigation of these Cross-cascaded attacks, Fraggle attacks and DoS variations within flow table overloading attacks include the unpredictable nature of network traffic, which makes it hard to distinguish between normal network activity and actual attacks on the network. The large volume of data in large networks creates problems in the detection system since it may not be able to detect the attacks or generate false alarms. Also, the wide range of networks and their configurations makes it impossible to develop universal detection algorithms that are flexible enough to work in all network architectures. Lack of visibility in the state and dependencies of the network infrastructure is an obstacle to proper anomaly detection in complex SDN environments with multiple controllers and switches. Another problem is balancing between precision and computational efficiency because constrained environments make it hard to use advanced detection algorithms. Resilience of the detection algorithm against attacks on the detection system is an issue that needs to be addressed. Therefore, an architecture of the 5G-SDN network with in-built security features would be a good choice to solve this problem. Consequently, the problem is compounded by the absence of standardized benchmarking and evaluation frameworks to allow for a comparison of the algorithms used for assessing the effectiveness of these detection techniques (Kazmi et al., 2023; Al-Fayoumi and Al-Haija, 2023; Moubayed, 2024; Yang et al., 2024; Kumar and Agrawal, 2023; Salahdine et al., 2023). SDN not only ensures centralized control but also opens up new vulnerabilities, such as distributed attacks (low-volume attacks) and flow table exhaustion. To address these challenges, the research develops an architecture that utilizes adaptive classification for precise attack detection, deep feature encoding for efficient representation learning and clustering for traffic grouping. Each architectural component was selected to address specific vulnerabilities associated with SDN, such as dynamic traffic behavior, scalability issues, and advanced attacks. By combining all these architectural components, a reliable, controllable, scalable, and effective SDN protection technique against current network attacks is achieved. Thus, Figure 1 shows a 5G SDN architecture that is tier-based and includes security functionalities. This figure illustrates the architecture of a 5G SDN network that is integrated with security functionality. The architecture consists of four planes: the 5G Wireless Plane, which stands for different user use-cases, such as immersive, tactical, ultra-reliable, seamless, ultra-dense, and massive connectivity.

The data plane enables communication between devices and implements resource policies through security mechanisms against attacks. Switches operating in the data plane are designed to be fast through special hardware to rapidly look up the entries in the forwarding tables and deliver packets at line-rate speed. Layer two Control Plane is responsible for network management with the help of the SDN controller that provides secure routing and traffic management. An application security framework exists in the Application Plane for threat monitoring and management purposes. The illustration depicts a strong multi-layered architecture of network management and security in 5G networks.

### 1.1 Motivation and core contribution

Unique strategies need to be applied in order to effectively identify and resolve any abnormalities during the flow table overloading attacks. Thus, this research makes the following contributions

- A new method for clustering to assign resources to switches when flow table overloading attacks are performed on a 5G-SDN network is proposed, while keeping necessary functions such as deep packet inspection and content filtering intact.
- A Deep dual-stream algorithm has been proposed, which will detect low-volume attacks in a 5G environment by analyzing the spatial and temporal traffic of the network.
- Deploys a DL-based approach, DCLSTM, to classify and mitigate various attack types with unknown attacks, improving the accuracy and robustness of intrusion detection systems by learning subtle spatial-infiltration markers in network traffic.
- Improves the detection accuracy of flow table overloading attacks and other evolving threats by introducing the Improved Chaotic Walrus Optimization (ICWO) algorithm, thus strengthening the security of 5G networks.

Hence, this study introduces innovative solutions to enhance 5G-SDN network security. Finally, the algorithm boosts intrusion detection accuracy and robustness against evolving threats.

### 1.2 Key objectives

The main objectives of this research are to improve the security and performance of 5G-SDN networks by tackling the issues presented by flow table overloading and other growing cyber threats. This involves developing a novel clustering strategy for dynamic resource allocation during attacks, creating a dual-stream system to precisely evaluate geographical and temporal network traffic data, and implementing a deep Learning-based CLSTM model to categorize and mitigate various attack types. Moreover, the research focuses on enhancing intrusion detection accuracy and ICWO, which will ensure effective protection against known as well as unknown intruders in the 5G environment. Therefore, some interesting technical research questions about the research topic have been analyzed below.

- How can dynamic clustering optimize resource allocation during flow table overloading attacks in 5G-SDN networks?
- What techniques effectively distinguish benign fluctuations from low-volume anomalies in 5G network traffic?
- How can DL models enhance detection and mitigation of diverse, unknown threats in 5G real-time scenarios?
- How can deep packet inspection and an advanced classification model strengthen 5G-SDN security while ensuring functionality?
- How does the Improved Optimization algorithm improve detection accuracy against evolving threats?

These research questions intend to solve the critical problems of secure 5G-SDN networks through exploring new solutions for dynamic resource allocation, attack detection, and network optimization. These questions will provide useful information for designing a security framework for 5G networks that can efficiently and effectively mitigate the mentioned problems. The structure of the research is organized in the following way. First of all, Section 1 provides an introduction to the research where the background information, problem statement, and research goals will be presented. Section 2 will be devoted to presenting a thorough literature review of 5G-SDN networks. In Section 3, the proposed methodology will be introduced; particularly, new methods of dynamically overcoming flow table overloading attacks will be discussed. Section 4 will be devoted to the description of the implementation and evaluation criteria of the proposed solution. Section 5 will be devoted to discussing the results and analysis of the proposed approach with a comparison to other existing approaches, as well as statistical analysis. Section 6 will contain the discussion of Practical Deployment. Conclusion and Future Directions will be provided in Sections 7 and 8, respectively.

## 2 Literature review

Han et al. (2024) applied different ML models like XGBoost, Random Forest, and SVM together with feature selection strategies to enhance intrusion detection on a number of benchmark datasets. Although the method produced effective detection within an acceptable calculation time, it is not fully validated against new or unnoticed threats. For the purpose of handling dynamic assault situations in 5G and beyond networks, Nezhadsistani et al. (2026) proposed a hybrid and explainable approach to intrusion detection that combines an Autoencoder with an XGBoost algorithm. While the proposed technique adds significant computational burden and provides insufficient capability for multi-class threat recognition, it managed to show good accuracy and reliability. In order to optimize DDoS attack detection in 5G network slicing environment, Behnam Farzaneh et al. (2024) developed a hybrid framework that consists of BiLSTM, CNN, ResNet, and transfer learning. While the proposed approach demonstrates promising results in terms of detection performance, its complexity prevents it from being implemented in edge conditions due to resource restrictions. In order to detect and mitigate DDoS attacks happening in SDN-based IoT architectures, Khedr et al. (2023) created a multi-layer ML framework with high precision and fast detection. Scalability and use of training data related to time in larger systems, however, are still problems. For more effective interpretation during intrusion detection applications, Azeez et al. (2026) used Graph Neural Networks along with multi-head attention and SHAP algorithms. The system fails to scale up in size and complexity of the network due to its high efficiency and speed. For identifying intrusions in virtualized 5G systems, Moubayed (2024) introduced a DL approach utilizing CNN and LSTM architectures. The framework worked effectively, achieving more than 99% precision on the 5G-NIDD dataset. Nevertheless, the algorithm faces difficulty when it comes to detecting unknown attack patterns.

Abdulqadder et al. (2020) developed an innovative method to prevent security attacks in a cloud of 5G networks backed up by SDN/NFV. The ML-IDP approach is comprised of five layers, including data acquisition, switches, intelligent controllers, domain controllers, and virtualization. The Four-Q-Curve algorithm is applied in the first layer for user authentication. Deep Reinforcement Learning is used in the IDP agent in the switch layer for avoiding flow table overloading attacks. In the layer of domain controllers, the packets are assigned to suspicious classes through the Shannon Entropy Function. Various security threats have been tested for the ML-IDP approach through NS3.26, but blockchain technology was required for certain security threats, such as host location hijacking, DDoS, flow table overloading, IP spoofing, and control plane saturation. Abdulghaffar et al. (2021) have recommended the application of SDN-based 5G core architecture in order to increase flexibility and manageability of the network. Comparison of the proposed SDN-based 5G architecture with the existing 5G architecture was performed based on the end-to-end latency. The results, depending on various parameters for various procedures, indicate that the proposed architecture provides a lower end-to-end delay than the existing one. However, further research on load balancing and scalability of the SDN controller is needed.

The researchers Yungaicela-Naula et al. (2021) proposed a modular and flexible SDN framework using various DL/ML models to detect DDoS attacks on the transport layer and application layer. In analyzing several methods of ML/DL, it was possible to understand which methods can be used effectively under specific attack conditions. This work analyzed ML/DL models using two different security data sets, CICdoS2017 and CICdoS2019. This work proved that the models perform better than 99% accurately in detecting unknown traffic (testing set). A simulated environment is created in this work using the Mininet network simulator and SDN controller. This work achieved a high accuracy rate of 95% in detecting application-layer DDoS attacks and above 98% for transport-layer DDoS attacks. The goal of this work is to evaluate and implement a better mitigation strategy.

The work of Alamri et al. (2021) studied the issues of security in Distributed DDoS attacks in SDN under 5G networks. This research considered the application of ML, especially the Extreme Gradient Boosting (XGBoost) algorithm, for the protection of the SDN controller. With the help of the combination of ML algorithms and adaptive bandwidth allocation, together with dynamic thresholds, this method allows effective identification and prevention of DDoS attacks and optimization of the operation of the network. XGBoost increases the accuracy of attack detection, decreases the CPU load, and lowers the energy consumption costs. Performance evaluation demonstrates the increase in security due to the reduction in the number of dropped packets and effective management of DDoS attacks. Nonetheless, the scheme is very sensitive to the correctness of the ML model and, therefore, requires substantial training data to function at its best. The dynamic threshold system could also introduce delays or inaccuracies under rapidly fluctuating network conditions, limiting real-time adaptability.

Andrade et al. (2024) study addressed the vulnerability of IoT devices in 5G networks to DDoS attacks, especially traditional intrusion detection due to challenges posed by General Packet Radio Service (GPRS) Tunnelling Protocol User Plane (GTP-U). The challenge of underperforming the system was addressed through a new proposal of an IDS framework by incorporating SDN, ML, and programmable switches through the P4 language. With the combination of the P4 switch and the ONOS controller, there is an improvement in the system’s performance in detecting and defending against the DDoS attack conducted by the malicious IoT devices. However, this research is dependent on a particular 5G testbed, which might not accurately represent the range of actual network conditions. Additionally, the approach focuses on GTP-U traffic, potentially overlooking other protocols that may be used in DDoS attacks within 5G networks.

Alshra’a et al. (2021) research introduced the Recurrent Neural Network (RNN) and LSTM to recognize and mitigate intrusion attacks. The model is evaluated using the InSDN dataset and shows promising results of higher accuracy of 0.98, precision of 0.978, recall of 0.99 and F1-score of 0.987. However, the limitations are that training time is longer for LSTM and identifying novel attacks or threats is a strenuous effort. Liu et al. (2024) proposed a gray wolf optimization algorithm and prediction model developed by integrating the LSTM network for designing a network security prediction system. The results obtained are MAE of 0.0237, RMSE of 0.0012, MSE of 0.0366 and mean absolute percentage error of 0.071. Although the proposed solutions achieved a lower rate of error value, they still need to enhance network security for specific attacks. Afroj et al. (2024) suggested three novel approaches to improve the precision of detecting DoS/DDoS attacks in SDN environments. One hybrid model, CNN-LSTM, achieves an accuracy of 98.51%, focusing solely on specific attacks rather than unknown ones. Jaber Alhilo and Koyuncu (2024) proved the model’s performance through a series of steps with a CNN-LSTM framework improved by SCA-TSO optimization to identify network traffic patterns of 99% precision.

To facilitate refining a ResNet-BiGRU model for intrusion detection across common datasets, Xia et al. (2024) presented a hybrid optimization technique utilizing PSO and GA. Despite achieving excellent precision, the method’s higher training complexity and lack of attention to SDN-specific attack scenarios are problems. Considering the target of minimizing packet loss while preserving effective traffic forwarding, Kong et al. (2024) created a lightweight defence mechanism to handle flow table overflow attack attempts in SDN. The technique has proven to be efficient but is only suitable for some types of attacks and should be subjected to further tests in a complex and dynamic network environment. An analysis comparing the performance of ML and DL methods in detecting DDoS attacks in the SDN framework has been presented by Hirsi et al. (2025). Even though the research contains useful information, it does not contain any real-time implementation details. A 5G network-focused intrusion detection system based on Transformers has been created by Harshdeep et al. (2025), which gave excellent results in terms of several metrics. However, the model is too computationally expensive and lacks interpretability for practical purposes. Table 1 shows the comparison of the current techniques with the results.

**Table 1:** Systematic comparison of related work methods.

References	Technique/model	Main objectives	Dataset used	Quantitative results	Research gaps
Moubayed (2024)	CNN + LSTM	Developed a DL pipeline for softwarized 5G networks' intrinsic intrusion detection.	5G-NIDD dataset	Accuracy—99.05%, recall—98.7%	Weak generalization to unknown attacks
Han et al. (2024)	XGBoost, RF, SVM	Enhanced IDS performance with ML and feature selection	InSDN, CICIDS2017, CICIDS2018	Time costs are 1.81 s, 5.78 s, and 6.70 s.	Limited zero-day attack evaluation
Nezhadsistani et al. (2026)	Autoencoder + XGBoost + XAI	Proposed an explainable hybrid IDS for dynamic 5G/B5G attacks	CICIDS2017+5G-NIDD + 5G	Accuracy—99.21%, F1-score—99.02%, AUC—0.998	High computational overhead; Lack of identifying multi-class attacks
Farzaneh et al. (2024)	BiLSTM + CNN + ResNet + Transfer Learning	Enhancing 5G networks' ability to identify DDoS attacks	5G NETWORK SLICING Testbed	Accuracy—97.8%, F1-score—97.5%	Not lightweight; poor edge deployment feasibility
Khedr et al. (2023)	FMDADM-Machine Learning-based DDoS mitigation	Constructed a multi-layer Machine Learning framework for SDN-IoT detection and prevention of DDoS.	SDN-based IoT Network simulation	Acc—99.79%, Prec—99.43%, recall—99.79%, F1-score—99.77%, Specificity—99.95%, False Positive Rate—0.21%, Detection Time—2.64 μs	Training timestamp and scalability issues
Azeez et al. (2026)	GNN + multi-head Attention	Enhanced intrusion detection with graph-based learning and used SHAP to give explainability.	5G-NIDD, NGIDS-DS	Accuracy—98.67%, detection rate—99.20%, F1-score—98.9%, latency—24.6 ms	Scalability challenges
Hirsi et al. (2025)	SDN DDoS detection	SDN-based DDoS classification and detection method	SDN DDoS datasets	RF, SVM, CNN, LSTM, and XGBoost models were compared; detection accuracy varied between 90% and 99%.	No practical implementation details
Harshdeep et al. (2025)	Standalone transformer	To improve the accuracy of DDoS detection in 5G networks, an IDS based on transformers was designed.	5G-NIDD	Acc—99.79%, Prec—99.76%, recall—99.72%, F1-score—99.74%, AUC—0.999	Limited explainability and high computational overhead
Xia et al. (2024)	PSO-GA + ResNet-BiGRU	Hyperparameter optimization for deep IDS models	KDD99, UNSW-NB15, CICIDS2017	Accuracy—99.36% on KDD99 and 99.21% on CICIDS2017	Higher training overhead; limited explainability, not specific to SDN attacks
Kong et al. (2024)	rDefender (Flow Table Overflow Protection Mechanism)	To prevent Flooding attacks, a robust, lightweight defence system is used.	SDN topologies in practice and scenarios of flow table overflow attacks	Stable mitigation performance against a variety of FTOA attacks was attained; packet loss was greatly decreased; and attack flow eviction efficiency was increased.	Restricted to particular sorts of flow table overflow attacks; unexplored scalability in ultra-large 5G-SDN environments
Alshra’a et al. (2021)	RNN + LSTM	Compared DL techniques for precise and effective threat identification in SDN systems	InSDN Dataset	Acc—98%, Prec—97.8%, recall—99% and F1 score—98.7% (DoS)	Evaluated only a few attacks (not multi-attack scenarios).
Liu et al. (2024)	Improved Grey Wolf Optimization (IGWO) + Neural Network	Improving network security scenario forecasting with IGWO-integrated neural networks with optimum parameter tuning	Simulated attack scenario	High accuracy, MAE of 0.0237, RMSE of 0.0012, MSE of 0.0366	Insufficient assessment of real-world datasets and insufficient validation for various cyberattack scenarios

### 2.1 Challenges and research gap

Based on the literature review, there are certain gaps that have been identified to improve security and resource management in 5G and SDN.

- The first problem relates to the optimization of network resource allocation, which can be solved through AI integration. The second one refers to the lack of scalability in SDN controllers because many currently available solutions cannot be scaled effectively in large, complicated networks (Abdulghaffar et al., 2021).
- Most of the current models demonstrate low adaptability in detecting new and zero-day attacks because most of them are designed and tested according to the known types of attacks (Moubayed, 2024; Han et al., 2024).
- Concerning the security aspect, it may be useful to integrate Blockchain technologies into SDN, NFV, cloud, and 5G networks as a way to improve the current security infrastructure because optimized methods of mitigation have not been tested yet. The main problem of securing 5G and SDN networks from the flow table overloading attacks is still relevant (Yungaicela-Naula et al., 2021; Alamri et al., 2021)
- Flooding attacks make use of an overwhelming number of flow entries that hinder the capability of switches in performing DPI and content inspection, allowing any kind of malicious data to pass without being noticed. Variability in network traffic makes it even harder to detect any low-volume attacks since current systems highly depend on static thresholds or statistics for this purpose (Andrade et al., 2024; Alshra’a et al., 2021).
- Some studies make it hard to distinguish between any kind of attacks and regular traffic flow variations, which leads to misdiagnoses (Liu et al., 2024). It is necessary to develop an adaptive solution with dynamic resource allocation and improved load balancing to defend against potential threats in large-scale 5G-SDN networks.
- The significance of complex models is still limited by scalability, particularly in large-scale and dynamic 5G/SDN environments (Khedr et al., 2023; Azeez et al., 2026). Many transformer-based and deep Learning methods are not sufficiently explainable, which reduces their transparency and dependability (Harshdeep et al., 2025; Xia et al., 2024).
- Some studies lack real-world implementation and validation and instead focus mostly on benchmark datasets (Hirsi et al., 2025). Some solutions are only applicable to specific types of attacks or situations, which restricts their broad use in a range of network conditions (Kong et al., 2024; Xia et al., 2024).

## 3 Proposed framework of Affinity Cohesive Clustering and Dual Info-Varcoder with ICWO-CLSTM for attack detection and mitigation

The problems with the above research can be handled using a novel hybrid model and also for detecting and preventing the attacks in 5G-based SDN networks, an innovative concept is introduced, “Affinity Propagated Cohesive Clustering and Deep Dual Info-Varcoder with Dense Convolutional Long Short-Term Memory with Improved Chaotic Walrus Optimization” in the data plane layer of the 5G SDN. The 5G network environment is deployed within the 5G organization layer, where the data plane is employed with switches. To solve the overwhelming number of switches during the flow table overloading attacks, the Affinity Propagated Cohesive Hierarchical Clustering (APCHC) technique is introduced, where Affinity propagation dynamically identifies clusters of switches based on pairwise similarities in resource usage, allowing for adaptive allocation of resources to clusters experiencing higher loads. This facilitates the efficient distribution of resources and ensures that critical functions like deep packet inspection (DPI) and content filtering are adequately supported across the network. Moreover, the application of Agglomerative Hierarchical Clustering helps to increase efficiency in resource allocation because it offers hierarchical clustering of the switches based on their resource usage patterns. Since the APCHC approach allows for dynamic adjustment to the changes in the network environment, it enables better performance of the flow table and avoids overload of the network.

To detect and defend against the attacks on flow table overloading attacks in the data plane layer, it is necessary to use DDST-IV in combination with RNN-LSTM. DDST-IV makes it possible to distinguish normal variation from possible anomalies by virtue of a thorough understanding of the nature of network traffic variation. DDST-IV consists of two streams that enable the analysis of both spatial and temporal features (Source IP and Destination IP addresses, MAC address, packet size, protocol type; packet arrival time, packet departure time, inter-arrival times, processing time and queueing time respectively). Analyzing these aspects of network traffic allows one to identify slight yet important variations in traffic patterns that are typical for attacks despite their low intensity. Using variational information encoder techniques, DDST-IV learns to encode the underlying structure of normal traffic patterns while accurately capturing anomalies that deviate from this learned distribution. Thus, DDST-IV, with its learning capability, effectively enhances the detection accuracy of security systems, reducing the risk of false negatives and enabling the timely identification of evolving attacks in network infrastructure.

To further identify and neutralize attacks such as Cross-cascaded (CC) attacks, Fraggle attacks (F), DoS Goldeneye attacks (DGY), DoS Slow Loris attacks (DSL), DoS Hulk attacks (DH), Brute force attacks (BF), and other unknown attacks, this research proposes the application of the densely connected convolutional LSTM (DC-CLSTM) network. By exploiting the potentialities of DL offered by the CLSTM network, DC-CLSTM removes all shortcomings associated with the current methods. Contrary to the LSTM that tracks irregular session durations and inter-packet intervals in the time domain, the convolutional layers in CLSTM detect any anomalies present in packet streams. This network is capable of identifying complex relations like irregular usage of ports and inconsistent source-destination relations, making it possible for the model to detect even tiny signals of penetration, including unusual protocol changes due to dense connectivity that makes it possible for features to be reused. Furthermore, through the extraction of information temporally and spatially from network traffic data, the model will be able to find small patterns of low-level attacks that cannot be detected by existing statistical methods. The use of CLSTM for the extraction of features and the sequencing of network traffic data enables the DC-CLSTM model to learn about the complicated relationship between data in the network. Consequently, such a model can be applied to classify and counterattack attacks regardless of their difference from the predefined levels or patterns.

**Figure 2:**
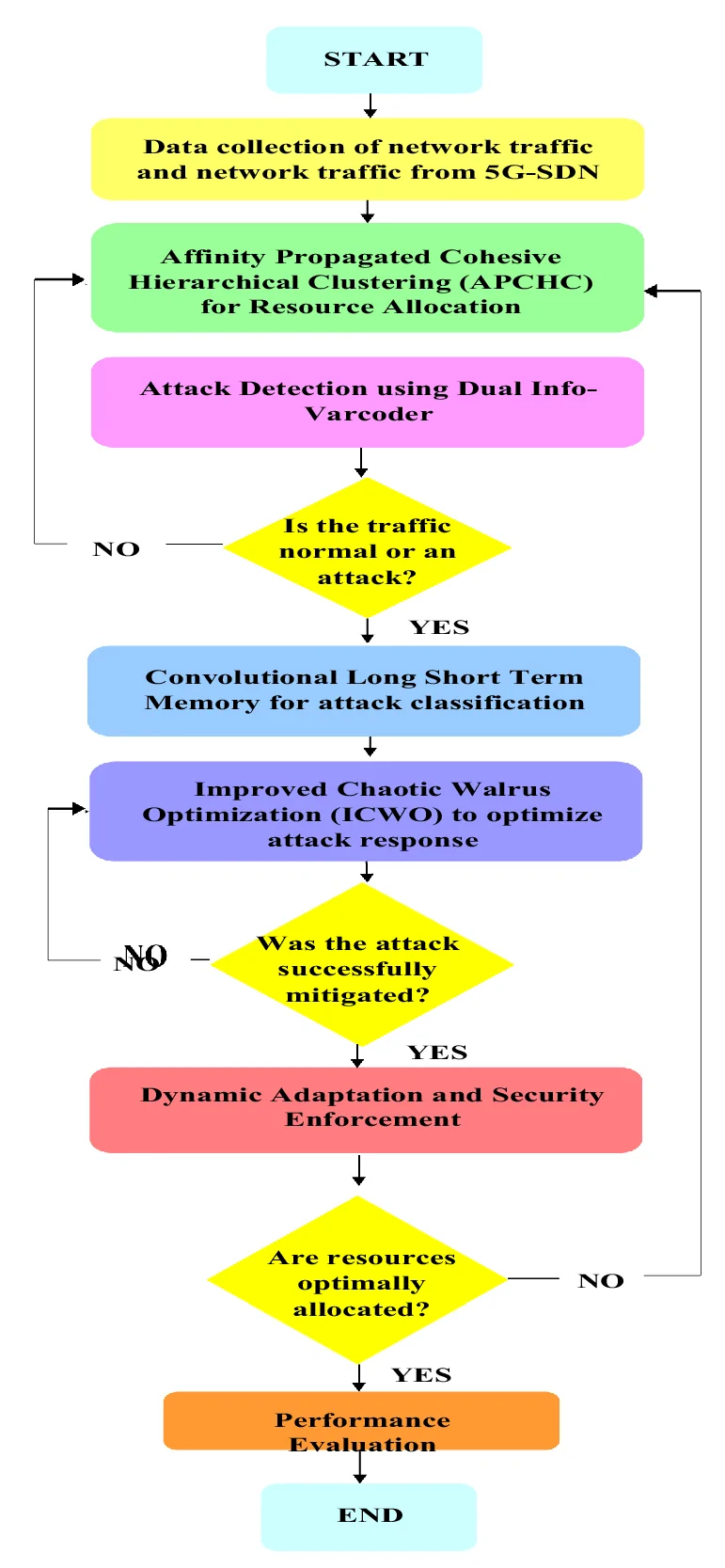
Workflow of APCHC and Dual Info-Varcoder with ICWO-dense CLSTM approach.

**Figure 3:**
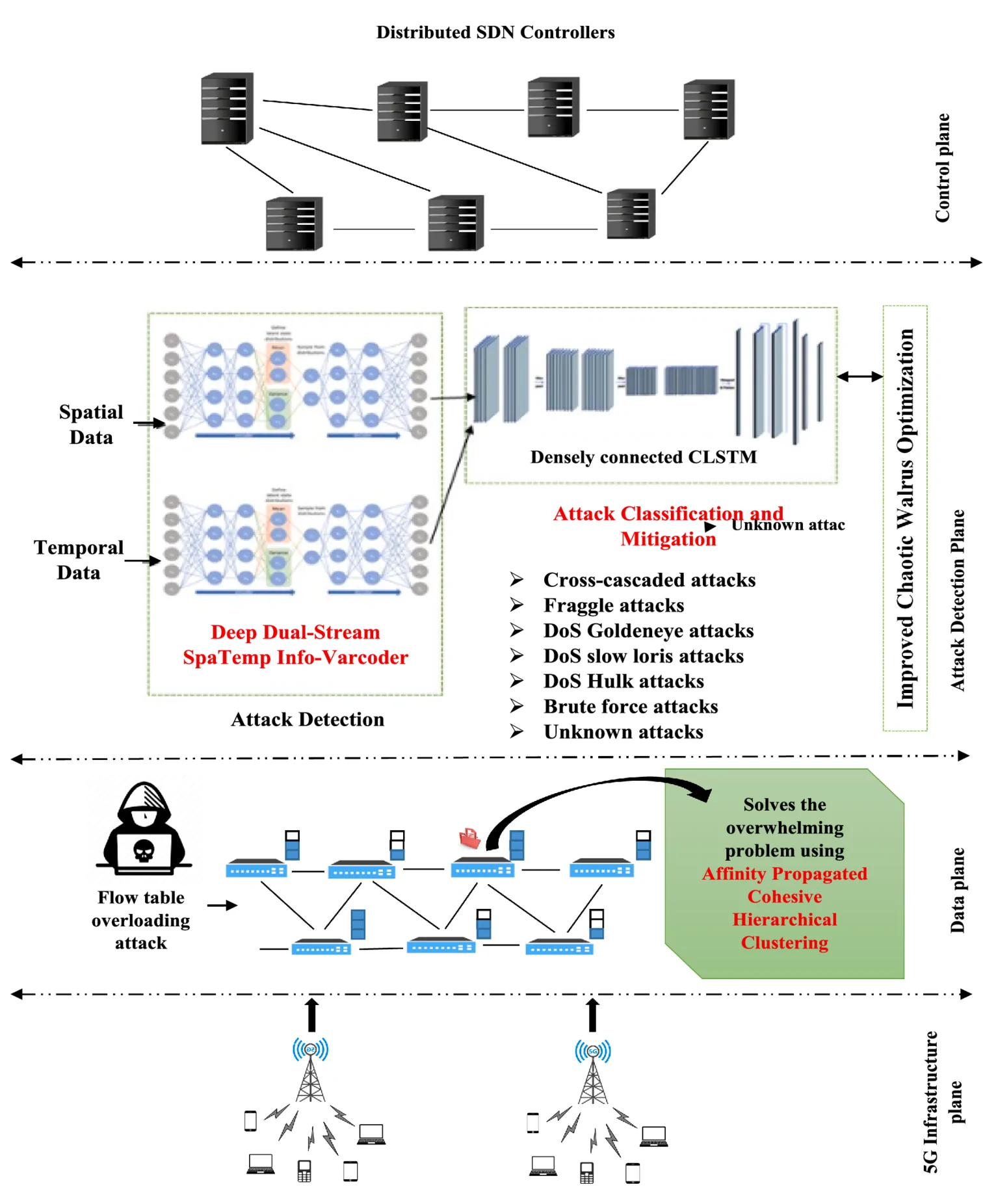
Framework of the proposed methodology.

DC-CLSTM increases the robustness of the infrastructure of the network because it becomes difficult for attacks with dynamic methodologies to occur, since the accuracy of detection and faster and more specific actions taken for the new attack detected by the system increase. The algorithm ICWO is utilized to optimize the system parameters after the detection and mitigation of the unknown attacks through the use of the DC-CLSTM system, as illustrated in Figure 2. This adaptive approach ensures the system remains effective against evolving attack tactics and changing network conditions. Hence, the total performance and security of the 5G-based SDN network are enhanced by this optimization process, which guarantees improved adaptation to network dynamics. Figure 3 represents the framework of the proposed model of the complete flow.

### 3.1 Advanced clustering for secure network management (APCHC)

Normally, switches are deployed within the data plane layer, where they are responsible for packet forwarding and flow table management. These switches are integrated with network surveillance tools to make real-time data acquisition and analysis possible. Integrated with OpenFlow to enable dynamic control of traffic based on real-time traffic patterns, these switches are updated periodically, where traffic monitoring tools collect network statistics such as flow rate, packet size, and packet drop rates. Data collection helps in attack detection and anomaly prediction. The flow rate *R_f_* for data packets is calculated using Equation 1 (Qureshi, 2024),


$$R_{f}=\frac{\Sigma_{i=1}^{n}P_{i}}{T}$$ (1)

**Figure 4:**
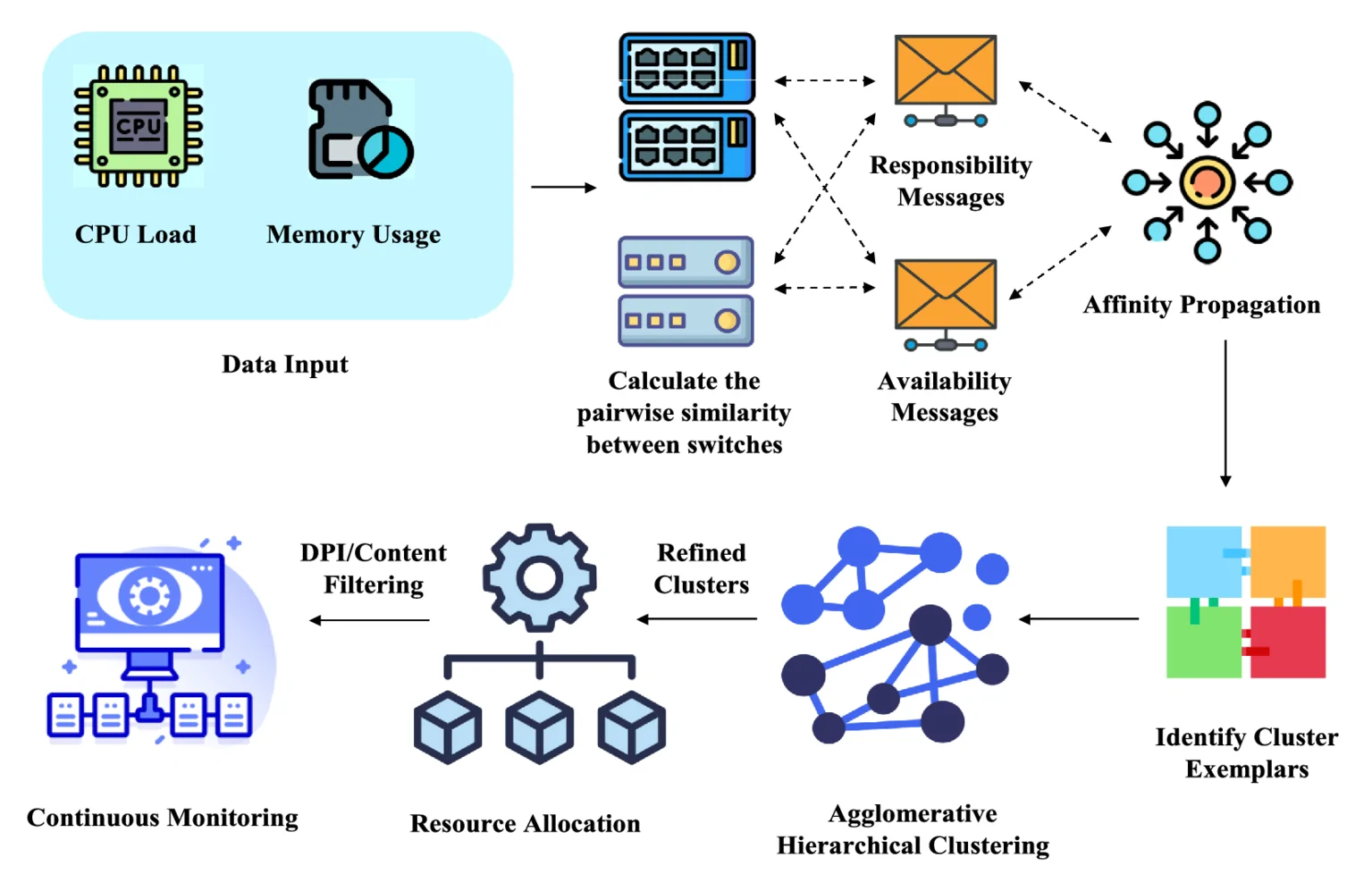
Mechanism of APCHC for resource management and clustering.

Where 𝑃_𝑖_ is the number of packets forwarded during time, *n* is the total number of flows, and *T* is the period for monitoring. Continuous monitoring tools then provide critical insights into traffic behavior that enable proactive anomaly detection. These insights validate that the network stays resilient even in the face of dynamic traffic patterns and potential threats. The integration of OpenFlow allows for real-time traffic adjustments, ensuring that network resources are allocated effectively. Such a system increases the efficiency of operations and also boosts the security features in the 5G network. The Affinity Propagated Cohesive Hierarchical Clustering (APCHC) algorithm is a sophisticated tool used to enhance security and resource utilization in network environments, as shown in Figure 4.

This technique begins with Affinity Propagation, which dynamically identifies clusters of switches based on pairwise similarities in resource usage, such as CPU load and memory utilization. This dynamic clustering allows for adaptive resource allocation to clusters that are experiencing higher loads, preventing overload and ensuring efficient resource distribution. APCHC technique dynamically identifies clusters of switches based on their resource usage (CPU load, memory usage). First, the clustering process calculates the pairwise similarity between switches. (*S*_*i*,*j*_) is based on the Euclidean distance between their resource usage. Equations 2–6 are used for mathematical modelling of integrated switch clustering applications (Qureshi, 2024).


$$S(i,j)=\sqrt{{\left({\mathrm{CPU}}_{i}-{\mathrm{CPU}}_{j}\right)}^{2}+{\left({\mathrm{Mem}}_{i}-{\mathrm{Mem}}_{j}\right)}^{2}}$$ (2)

Where CPU*_i_* and Mem*_i_* indicate the CPU and memory use of switch *i*, CPU*_j_* and Mem*_j_* represent the CPU and memory usage levels of switch *j*. Affinity Propagation is then applied to determine the cluster exemplars (representatives), which are the switches with the highest combined responsibility and availability scores for each group. Two messages are exchanged between each pair of switches. Responsibility Message R(i,k) determines how suitable switch *k* is to act as the exemplar for switch *i*:


$$R(i,k)=S(i,k)-\text{kmax}\;\{A(i,k^{\prime })+S(i,k^{\prime })\}$$ (3)

Where *A*(*i*, *k*’) is the availability score for switch *k*’, Availability Message *A*(*i*, *k*’) determines how likely it is that switch 𝑖 should belong to the cluster of switch 𝑘 to become an exemplar for that cluster.


$$A(i,k)=\min\;(0,R(k,k))+i\sum \max(0,R(i^{\prime },k))\;$$ (4)

These two messages allow switches to communicate and eventually agree on cluster exemplars. After initial clusters are formed, Agglomerative Hierarchical Clustering is employed to merge clusters based on their resource utilization patterns, refining the clusters for optimal resource distribution. The cohesion score of cluster C(*k*) is determined as the mean value of intra-cluster similarities.


$$C(K)=\frac{1}{|C_{k}|}\sum_{i,j\in C_{K}}S(i,j)\;$$ (5)

Here, 𝐶_k_ is the set of switches in cluster 𝑘, *S*(*i*,*j*) is the similarity between switches 𝑖 and 𝑗. The clustering process is done in iterations to ensure maximum cohesion of the clusters, leading to improved load balancing and network operation. As a result, APCHC is able to make dynamic adjustments to resource allocation with respect to changes in network requirements. Through this adaptive nature, APCHC eliminates the problem of flow table overloading, improves network efficiency and security by maintaining smooth traffic flow and efficient resource management. The resource allocation *R*_alloc_(*k*) for each cluster 𝑘 is in proportion to its cohesion measure relative to the total system resources. The dynamic resource allocation is done using the clustering process, allowing functionalities like deep packet inspection (DPI) and content filtering.

The resource allocation $R_{\text{alloc}}\;(k)$ for each cluster 𝑘 is given by


$$R_{\text{alloc}}\;(k)=\frac{C(K)}{\sum_{k\prime \in \mathrm{All}\;\text{clusters}}C(k^{\prime }\;)}\times \text{Total Resources}$$ (6)

Where *C*(*K*) represents the cohesion of cluster *K*, Total Resources represent the total system resources. This will ensure that clusters with more loads have enough resources to achieve optimum performance. Monitoring of network traffic and resource utilization is done continuously to ensure system stability and to avoid overflow in the flow tables. This is done in real time to adjust clustering and resource allocation according to the changing requirements of the network.

### 3.2 Deep dual-stream spatial–temporal information varying autoencoder (DDST-IV)

**Figure 5:**
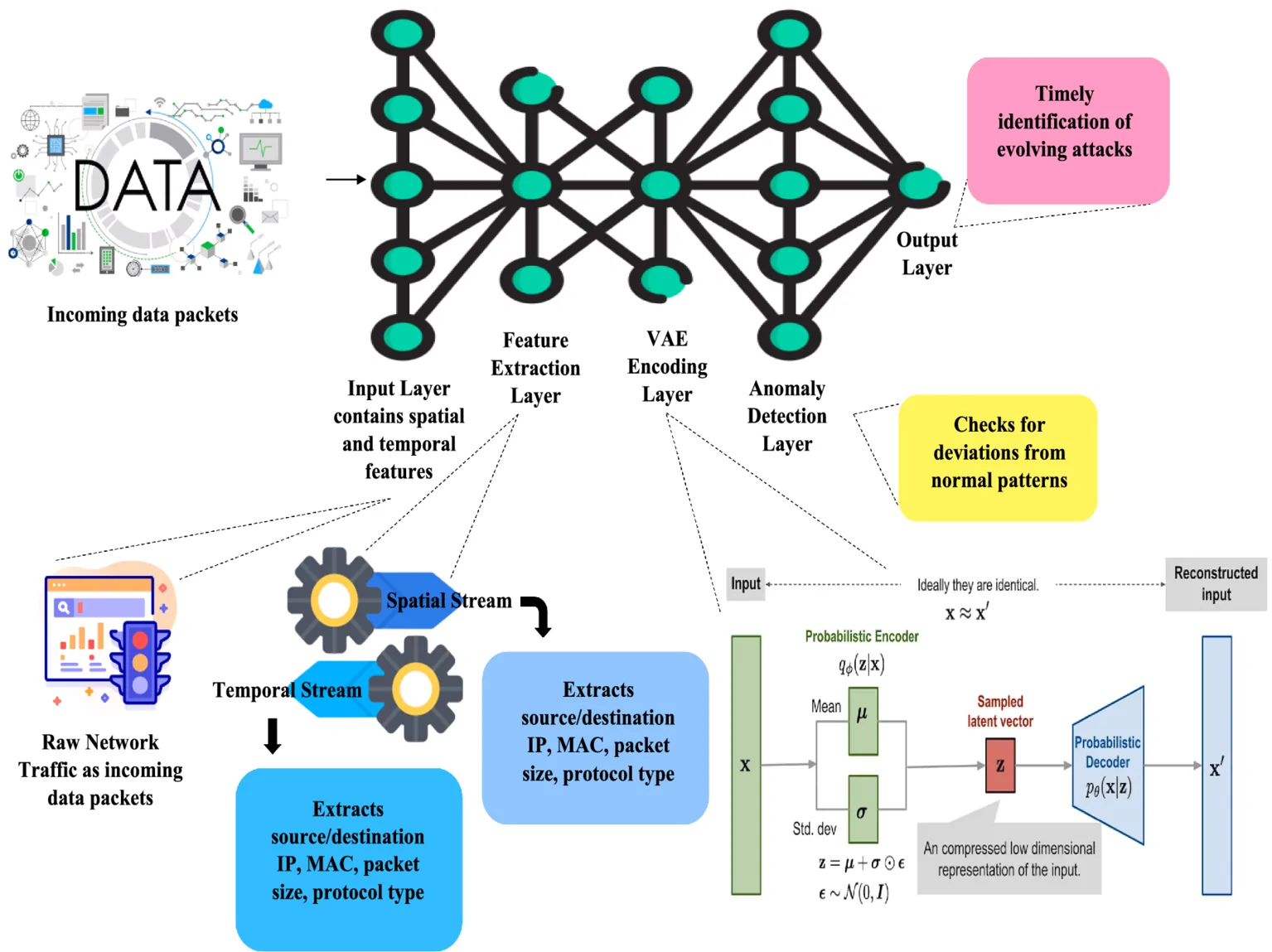
Layered architecture of DDST-IV for attack detection.

For attack detection, the DDST-IV is deployed alongside the RNN-LSTM. DDST-IV utilizes a dual-stream approach to analyze both spatial aspects (e.g., Source IP and destination IP addresses, MAC addresses, packet size, and protocol type) and temporal aspects (e.g., packet arrival and departure times, inter-arrival times, processing time, and queueing time) of network traffic. Figure 5 represents the diagram of attack detection using DDST-IV.

DDST-IV uses a variational autoencoder (VAE) approach to learn normal traffic patterns and detect deviations that indicate potential anomalies. This encoding process can be represented by the mathematical Equation 7 (Abdulghaffar et al., 2021).


$$z∼q_{\phi }(z|x)=N(z,\mu \phi \;(x),\sigma_{\phi }^{2}\;(x))$$ (7)

Where $q_{\phi }(\mathrm{zx})$ is the variational posterior distribution, *x* represents input traffic data, *z* is the latent space capturing normal patterns, and *μ_ϕ_*, $\sigma_{\phi }^{2}\;$ are the learned parameters from the traffic distribution. This allows the system to encode normal traffic behavior while capturing deviations, signalling potential anomalies in Equation 8.


z∼μϕ(x)+σϕ(x)·ε,ε∼=N(0,I) (8)

Where, ε is Random noise sample drawn from the normal distribution. N(0,I) is the standard normal distribution. Next, using the latent distribution, the decoder reconstructs the data input using Equation 9:


$$p^{\theta }(x|z)$$ (9)

where $p^{\theta }$ is the probability of reconstructing *x* from *z*. The covariance matrix of residuals is constructed using typical traffic patterns, and the reconstruction error is calculated using a Mahalanobis distance metric. This makes it possible to identify structured abnormalities by identifying inter-feature connections. Samples that exceed a predetermined threshold are categorised as attacks and mitigated by SDN-based flow control techniques.

The Mahalanobis reconstruction error is shown by $D_{\text{reconerror}}(x)$ in Equation 10,

$$D_{\text{reconerror}}(x)={\left(x-\hat{x}\right)}^{T}\Sigma^{-1}(x-\hat{x})$$ (10)

Where, $D_{\text{recon}}(x)$ is the reconstruction Mahalanobis distance of input sample x , $\hat{x}$ is the reconstructed or estimated version of x , $\Sigma^{-1}$ denotes the inverse covariance matrix. Based on the detection rule in Equation 11, if the reconstruction error is below the threshold, the sample is denoted as normal; otherwise above the threshold, the sample will be an attack.

$$\hat{y}=\{\begin{array}{c} 0,D_{\text{reconerror}}(x)\leq T\;(\text{Normal})\\ 1,D_{\text{reconerror}}(x)>T\;(\text{Attack}) \end{array}$$ (11)

where *T* is the threshold value that is used to distinguish between attack and usual traffic; normal traffic is indicated by a lower reconstruction error, while attack traffic is indicated by a greater reconstruction error. The threshold parameter T used during attack decision and optimization stages is selected using a trial-and-error approach by evaluating multiple threshold values under different traffic conditions and selecting the value that produces the highest detection accuracy with the minimum false positive rate. This adaptive threshold selection method enables the framework to achieve consistent detection of intrusion traffic despite the changing 5G-SDN traffic. Mitigation strategies have been introduced to ensure minimal effect on the detected attack traffic when the reconstruction error becomes greater than threshold *T*. These include the identification of anomalous traffic, isolation of suspicious traffic flows and blocking of malicious packets. In order to prevent further unauthorized access and network congestion, traffic filtering and rate limiting have also been applied. Once the mitigation strategy is completed, the system is still able to monitor the traffic to ensure that the reconstruction error returns to normal, hence indicating successful completion of the mitigation and resumption of normal network operations. The thorough analysis made by DDST-IV enables it to differentiate between benign variability and potential anomalies. DDST-IV is able to learn normal traffic patterns through variational information encoding while detecting any deviation that could be an indicator of attacks. DDST-IV is therefore capable of detecting anomalies, including cases of low volume malicious traffic. This will enhance the accurate detection of attacks and reduce false negatives in the network environment.

### 3.3 Enhanced attack classification and mitigation with a dense CLSTM architecture

**Figure 6:**
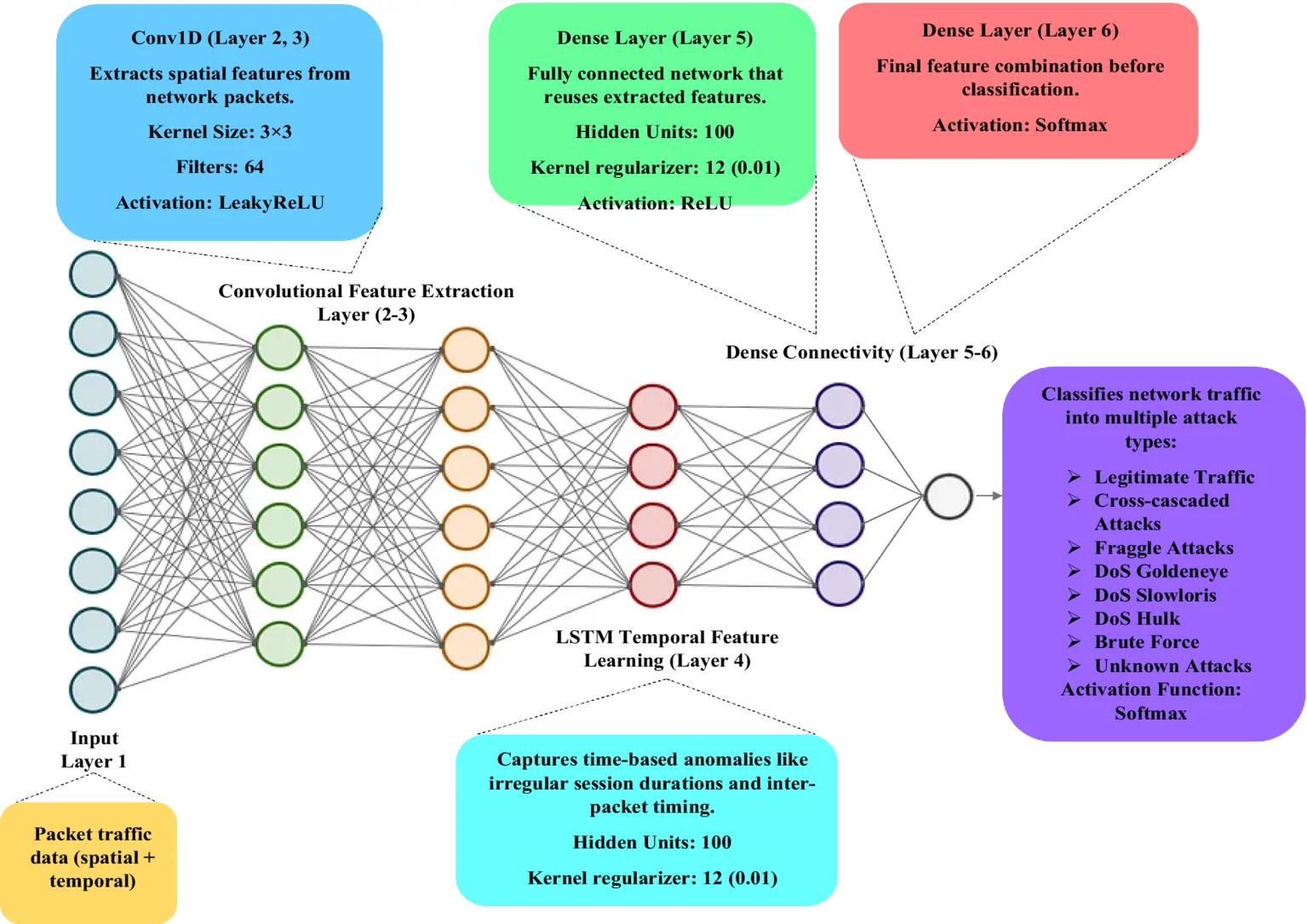
Design of DC-CLSTM for the efficient attack classification.

The DC-CLSTM neural network is applied during the classification and mitigation stages to improve intrusion detection and response. It combines dense connections with CLSTM neural networks to solve the shortcomings of the conventional methods, with a diagrammatic presentation of the architecture in Figure 6. The convolutional layers in the DC-CLSTM network are used to detect spatial characteristics such as anomalous patterns of packets, whereas the LSTM layers model temporal sequences in order to identify anomalies in session time durations and packet intervals. The dense connection in the DC-CLSTM network promotes reuse of extracted features and improves gradient flow to help identify subtle signs of network intrusion, such as protocol transition or atypical port usage. The DC-CLSTM network categorizes network traffic according to the type of attack. Based on analysis of the learning patterns, DC-CLSTM is able to distinguish between normal network traffic and malicious activities, even for those attacks that deviate substantially from the previous pattern or threshold values. The architecture of the DC-CLSTM detects both spatial and temporal features of network traffic data and can, therefore, be very useful in detecting low-volume attacks similar to legitimate traffic behavior. In this case, the convolutional layers of the CLSTM network deal with detecting the abnormal packet pattern by utilizing spatial features of network traffic, whereas the LSTM layers (Al Razib et al., 2022) take into account the temporal dynamics in terms of abnormal session duration or packet time intervals to find abnormalities. A densely interconnected network is used in order to facilitate learning through reusing the features and, hence, detect unknown markers such as unusual protocol switching and normal port behavior. Through modelling complex relationships, including inconsistencies in the mappings of source to destination addresses, the network is able to differentiate the malicious behaviors from the benign behaviors and improve the detection accuracy.

The function of an LSTM that models sequential dependencies is given in Equation 12.

$$h_{t}=\text{CLSTM}(x_{t},h_{t}-1\;)$$ (12)

With " $x_{t}$ " being the input at time " t ", and " $h_{t}$ " being the hidden state at time " t ", which captures information about previous traffic patterns to detect any anomalies. This process ensures that the false negative rate is reduced, making it possible to detect any attack or evolving attacks that include low-level malicious traffic. Mitigation actions are then carried out based on the classification results to mitigate the damage of the cyber threats. Changes are made to network policies and resource allocations accordingly to counter the threats. The quick and accurate mitigation makes the network infrastructure resilient to attacks. With the help of DC-CLSTM’s ability, the detection is improved even further through the use of ICWO.

### 3.4 Improved Chaotic Walrus Optimization for Effective Attack Detection (ICWO)

The ICWO algorithm is a sophisticated optimization algorithm used to optimize complex and highly dimensional problems in an efficient and effective manner. In the following section, the ICWO algorithm is discussed from the perspective of foraging dynamics and cooperative adaptability in walruses in the context of a chaotic environment. ICWO (Geng et al., 2024) uses chaotic maps to dynamically adjust the trade-off between exploration and exploitation in the problem space. This results in the capability of the algorithm to solve complex problem landscapes through efficiently escaping local optima while converging towards global optima. Through the process of dynamic parameter tuning and application of chaos theory, the ICWO algorithm is capable of solving problems in an efficient manner. Lastly, the following algorithm uses an optimization algorithm that tunes detection thresholds, selects features, and allocates resources in order to achieve optimal parameter convergence without getting stuck at local optima. The optimization algorithm described below improves detection efficiency, minimizes false positives, and optimizes resource allocation.

ALGORITHM 1 ICWO Algorithm for Efficient Attack Detection.
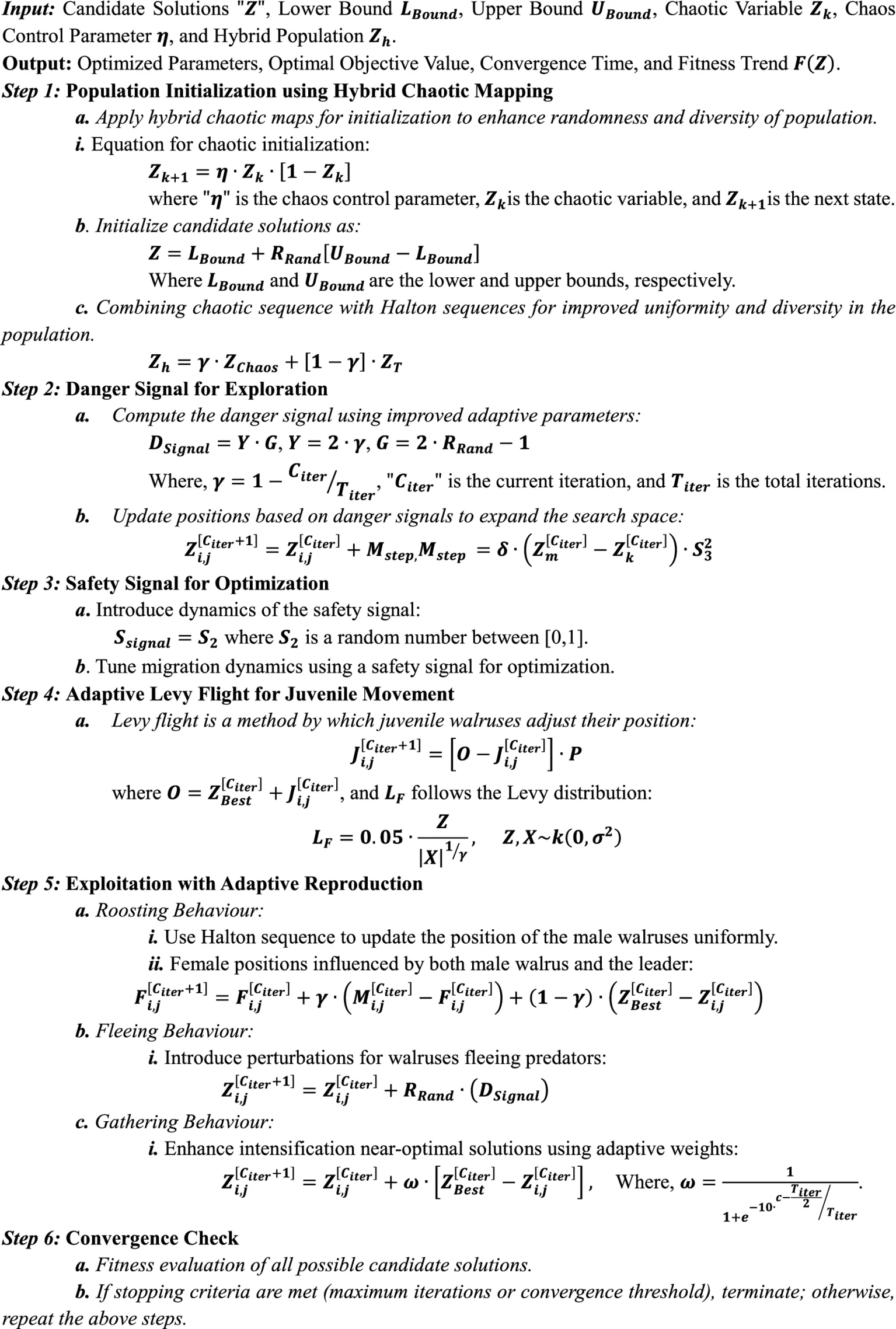

Here, Algorithm 1 demonstrates the ICWO Optimization process. In this case, the integration of the ICWO algorithm involves the utilization of WO (Walrus Optimization) through an analysis of Chaos Theory, Levy flights, and adaptation in order to optimize the exploration, exploitation, and convergence of the network. First, the algorithm starts with the initialization of the population, where hybrid sequences of chaos and Halton are used in order to achieve diversity. Exploration will be done using adaptive danger signals, while migration will be enhanced using safety signals. The juvenile walrus updates their positions through Levy flights in order to increase global search while exploiting the adaptive behaviors of roosting, fleeing, and gathering.

The complex, multi-modal and high-dimensional optimization problems in the context of threat detection using the SDN have led to the necessity for proposing the ICWO algorithm due to the fact that the current metaheuristics are often faced with premature convergence and lack of diversity. The Walrus Optimization Algorithm uses a chaos-based search mechanism to solve such problems. The property of ergodicity, randomness and the non-repeating nature of the chaotic system contribute to improving the diversity of the population and better examination of the search space. This particular property is important since discriminative attack patterns are usually hidden in the high-dimensional traffic space and cannot be found via local search. In the early stages, the chaotic algorithm allows better exploration of the global space as a result of the even distribution of possible solutions in the search space. It focuses on the exploitation of the promising areas as the search continues with the purpose of enhancing the solutions as well as accelerating the process of convergence. It is highly beneficial in the context of SDN attack detection because it improves feature selection and parameter optimization. As a result of this, there is better detection accuracy, reduced feature redundancy and lower computation costs. Thus, the proposed overall algorithm provides adaptive security in 5G-SDN networks through spatial–temporal analysis and optimisation-driven response mechanisms.

## 4 Implementation and evaluation criteria

### 4.1 Dataset selection and description

In order to provide a comprehensive and objective assessment of the performance of the proposed SDN-based attack detection and mitigation framework, the proposed model is evaluated using both artificial and existing benchmark datasets. For creating a dataset that can illustrate typical SDN architecture and multiple kinds of attacks, a custom dataset is developed with the help of the NS-3 network simulation tool and implemented using MATLAB. The use of a simulated dataset enables controlled testing and proper simulation of the network dynamics under different conditions. In addition to the custom dataset, public datasets including the InSDN Dataset, CICIDS2017, NSL-KDD and the ULAK dataset are used to validate the generalization capabilities of the proposed framework.

#### 4.1.1 5G SDN dataset generation and specification

The proposed model is based on the data obtained from Matlab SDN software, while a network simulation environment was designed through NS-3 Tool where the network behavior was simulated for a 5G SDN network exposed to seven kinds of cyberattacks such as Cross-Cascaded, Fraggle, DoS Goldeneye, DoS Slow Loris, DoS Hulk, Brute force, and Infiltration Attacks. The simulation setup consists of a 5G core network, SDN controllers (Ryu Controller), base stations (gNB), legitimate users (UE), and attack traffic generators. The Ryu SDN controller is used to dynamically regulate traffic flows in the NS-3 simulator, which is set up to simulate a realistic 5G-enabled SDN environment. To monitor network behavior, various traffic intensities were used to simulate attacks. Key traffic information, including packet count, flow duration, data rate, session concurrency and number of concurrent flows, which simulates large-scale attacks affecting SDN flow tables, was gathered from switches and the controller throughout the simulation, which are crucial factors affecting how traffic behaves in SDN-based 5G networks. Packet count was the primary parameter used to measure traffic intensity. The simulation provides variability in traffic patterns through a systematic manipulation of such parameters to enable the modelling of both low-rate and high-intensity attacks. To train the hierarchical clustering and DL algorithms, the resulting packet-level data must accurately represent SDN behavior, including flow rule dynamics, congestion impacts, and temporal fluctuations.

Dedicated malicious nodes create attack traffic, which is then injected into the network among authentic user traffic. Traffic patterns show different features in terms of inter-arrival time, flow duration and packet rate, depending on the type of attack. DoS Hulk and Goldeneye are examples of high-rate attacks that produce bursty traffic that clogs SDN switches and overwhelms the controller with flow rule requests. On the other hand, low-rate attacks like infiltration and Slow Loris sustain periodic or continuous connections, impacting resource utilization without appreciably raising bandwidth consumption. A crucial component of SDN vulnerability is that attack traffic causes unnecessary flow setup requests, which raise control plane overhead. Furthermore, based on the severity of the attack and the distribution of traffic, the 5G-specific elements, such as high data rate connections and latency-dependent communication between UE and gNB, are affected differently. In general, the traffic flow depiction shows how various attack scenarios are methodically introduced under a controlled environment and spread throughout the 5G-SDN environment, serving as the framework for a reliable feature extraction and training models.

To guarantee dataset reliability and minimize unpredictability, each attack scenario has been performed more than 200 iterations using different traffic parameters. The collected packet count values and other parameters were exported to MATLAB, where additional features like traffic variance, flow duration, inter-arrival time, throughput, packet loss rate, and entropy-based metrics were generated through feature engineering. The dataset named 5G SDN dataset comprises 1,18,347 samples of which 78,113 (66%) are normal samples and 40,234 (34%) are attack samples that includes Cross-cascaded (CC—6,481), Fraggle (F—5,676), DoS Goldeneye (DGY—6,386), DoS Slow Loris (DSL—5,467), DoS Hulk (DH—10,024), Brute force (BF—4,200), and Infiltration attacks (I—2,000). This dataset consists of 77 extracted features, which are further categorized into 54 for spatial features, which include structural and packet-based characteristics like Dest Port, Total Forward Packets, Flow Bytes/s, Packet Length Mean, and flag counts (FIN Flag Count, SYN Flag Count, etc.) and 23 for temporal features, which focus on timing-related traffic aspects such as Flow Duration, Flow IAT Mean, Fwd IAT Total, and Idle Min. A subset of sample feature values for typical traffic and seven attack class groups is presented in Table 2. With no missing values, the dataset is well-structured for attack detection and security analysis. In order to avoid the problem of sample bias and retain the natural distribution of data, stratified splitting was applied, making sure that the test will represent real SDN traffic situations. For the purpose of model validation, the data is split into 80% train (62,490 (52.8%)-normal, 32,187 (27.2%)—attack) and 20% testing (15,623 (13.2%)—normal, 8,047 (6.8%)—attack) sets, making sure that the proportion is kept properly for the robust detection of attacks in 5G-SDN networks. Class weights were applied during the training phase, making sure that the minority (attack) class had more weight. This helped to improve the ability of the model to detect attacks and reduce false negatives.

**Table 2:** Sample dataset feature values of the generated 5G-SDN dataset

Dest port	Flow duration	Total FwdPkts	Total BwdPkts	FwdPkt length Max	FwdPkt length Min	BwdPkt len Max	BwdPkt len Min	SubflowFwdPkts	SubflowBwdPkts	Fwd IAT Max	Bwd IAT Max	Label
80	120,000	400	389	1,500	64	1,400	60	400	389	3,000	2,800	Normal
53	90,000	385	64	512	60	500	58	385	64	1,500	1,400	CC
389	100,000	390	65	520	62	510	60	390	65	1,300	1,200	CC
139	100,000	721	120	1,024	64	200	50	721	120	500	450	Fraggle
139	90,000	712	119	1,100	70	220	55	712	119	450	400	Fraggle
53	90,000	580	97	1,500	100	300	60	580	97	200	180	DoS Goldeneye
53	100,000	584	97	1,450	95	280	58	584	97	220	200	DoS Goldeneye
80	150,000	1,007	168	820	65	200	50	1,007	168	10,000	9,000	DoS Slow Loris
443	100,000	796	133	1,500	100	550	65	796	133	100	90	DoS Hulk
465	95,000	75	65	1,200	70	1,050	60	75	65	2,500	2,400	Brute force
80	100,000	459	77	1,300	80	1,200	75	459	77	4,000	3,800	I attack

The simulation-generated dataset’s primary objective was to present a controlled and reliable environment that could simulate 5G-SDN-specific traffic behaviors, controller-level interactions, and emerging attack scenarios that are presently underrepresented in publicly accessible benchmark datasets, rather than to replace real-world datasets. Simulation is driven by the need to concurrently represent SDN control-plane behavior, 5G-specific network features, and new attack scenarios. The proposed simulation approach allows the development of SDN-aware and 5G-oriented attacks, including previously unforeseen attack patterns that directly impact controller-switch interactions, flow management, and network resources, in contrast to traditional benchmark datasets. The generated dataset, which originates from packet-level network interactions and controller-switch exchanges that mimic real SDN operational behavior, is in contrast to synthetic datasets created only on the basis of statistical assumptions. The simulation incorporates heterogeneous traffic situations, fluctuating traffic intensities, flow-rule updates, congestion effects, and temporal traffic variations in order to replicate actual network dynamics.

The generated 5G-SDN dataset is intended to simulate actual network conditions in a 5G environment with software-defined networking (SDN) enabled. Scalability, controlled testing, and the addition of new attack behaviors that might not be adequately captured in current datasets are all made possible by this synthetic dataset and enabling controlled evaluation of diverse attack scenarios in 5G-SDN networks. The synthetic dataset is adequate to train and evaluate the proposed algorithm because it includes both benign and malicious traffic patterns that are pertinent to 5G-SDN networks.

#### 4.1.2 Benchmark datasets (InSDN, CICIDS2017 and NSL-KDD datasets)

In addition to the generated dataset, the external benchmark validation was done using the InSDN, CICIDS2017 and NSL-KDD datasets. These publicly datasets are used as a benchmark dataset to verify the robustness and generalization ability of the proposed framework. It includes identified network traffic information collected through SDN configurations. The InSDN dataset is a large-scale, realistic network traffic dataset. There are 343,939 network traffic samples, of which 68,424 are normal samples (19.90%) and 275,515 are attack samples (80.10%). These fall into three groups: Open vSwitch (OVS) (data-plane attacks), Metasploitable-2 (control-plane attacks), and normal traffic. The Metasploitable-2 group comprises 136,743 samples. DoS (52,471), DDoS (48,413), Probe (36,372), brute-force (1,110), web attacks (192), and Botnet (164) account for 138,772 samples (40.34%) in the OVS group. The dataset is ideal for realistic SDN setups since it offers 80 features arranged into 56 clusters that capture multi-layer and multi-protocol traffic characteristics. Another widely used for intrusion detection research, CICIDS2017 is a massive, labelled dataset with realistic, typical, and varied attack traffic. Approximately 2.83 million network flow records, comprising 557,646 attack samples and 2,273,097 normal (benign) samples, make up the CICIDS2017 dataset. DDoS, DoS (Hulk, Goldeneye, Slow Loris, SlowHTTPTest), brute-force (FTP/SSH), botnet, web attacks (XSS, SQL injection), infiltration, and port scanning are among a variety of attacks. More than 80 features that reflect flow-based network characteristics are included. An extended version of KDD Cup 1999, which is frequently used for intrusion detection research, is the NSL-KDD dataset. It has 125,973 samples, tagged network traffic data with 41 features, and different attack variants, including DoS, Probe, U2R, and R2L. The research ensures the reliability and effectiveness of the 5G-SDN attack detection model by using these datasets to create a realistic evaluation framework, where the benchmark dataset offers real-world validation and the generated dataset facilitates scenario-specific testing for specific SDN data plane and control plane attacks in controlled experimentation.

### 4.2 System design and operational flow of a 5G security framework in SDN

The simulation results are given below, and this work was completed on the Python working platform using the following system specification.

**Table:** 

Platform (IDE):	Python (Spyder)
OS:	Windows 10
Processor:	64-bit Intel Processor
RAM:	8 GB RAM

The implementation uses Scikit-learn for clustering (Affinity Propagation and Agglomerative Clustering) and NumPy and Pandas for data preprocessing. The dual-stream variational encoder and Conv1D–LSTM model are constructed and trained using TensorFlow and Keras. Matplotlib and Seaborn are used for performance analysis and visualization.

#### 4.2.1 Data preprocessing

Data quality, uniformity, and applicability for the proposed 5G-SDN attack detection framework are guaranteed by the data preprocessing stage. After eliminating duplicate, incomplete, zero-duration, and noisy records, the NS-3-produced dataset’s initial sample count was reduced by roughly 20–30%, resulting in a final dataset with 1,18,347 samples and 77 features. Identifiers, redundant, and low-variance properties were eliminated to refine the features after outliers were identified and capped. Min–Max scaling was used to normalize all numerical features in order to guarantee consistent feature contribution. Where ‘*a*’ is an original attribute value and $a_{\max},a_{\min}$ are the maximum and minimum values of that feature is given in Equation 13. Because it speeds up convergence and enhances numerical stability, this phase is especially crucial for DL models.

$$a_{\mathrm{norm}}=\frac{a-a_{\min}}{a_{\max}-a_{\min}}$$ (13)

For efficient multi-class categorization, attack classes were specifically divided and encoded as Normal (0), Cross-cascaded (1), Fraggle (2), DoS Goldeneye (3), DoS Slow Loris (4), DoS Hulk (5), Brute force (6), and infiltration (7). To maintain class distribution and ensure reliable model training and assessment, the dataset was finally divided using a stratified 80:20 method.

#### 4.2.2 Hybrid optimized deep learning system architecture and training configuration

The proposed framework uses an architecture designed for complex SDN intrusion detection that is functionally reasonable. First, using APCHC to uncover the latent traffic structure and relationships between network flows, the goal is to increase feature discriminability and attack class separability. Second, by extracting complementary statistical and semantic representations, the Deep Dual-Stream Info-Varcoder makes it possible to learn deeper features and identify low-frequency and stealthy attacks more successfully. Third, the Densely Connected ConvLSTM enhances the learning of long-range attack behaviors by capturing extensive spatiotemporal relationships while encouraging effective feature reuse and robust gradient propagation. Lastly, Improved Chaotic Whale Optimization (ICWO) improves convergence, resilience, and generalization by optimizing model parameters and hyperparameters. Despite being computationally more complicated than traditional ML and DL models, the architecture intelligently makes use of this complexity to deliver better detection performance, accuracy, and robustness against an extensive range of novel and unknown SDN threats in 5G networks. Since these functionalities are non-overlapping, eliminating any component affects a crucial stage of the detection pipeline.

Using pairwise similarity generated from resource-related and behavioral variables, the Affinity Propagation (AP) method first divided the 300-switch SDN topology into 131 clusters. Due to variations in switch characteristics and traffic behavior, the resulting clusters differed in size. Five higher-level clusters with 111, 7, 12, 151, and 19 switches, respectively, were created by applying Agglomerative Clustering to combine similar AP clusters, as shown in Table 3. While the smaller clusters show unique or possibly abnormal switch activities, the concentration of switches within Clusters 0 and 3 implies prevailing behavioral patterns. The mapping of sample instances to Affinity Propagation (AP) clusters, Agglomerative (Agg) clusters, and Switch IDs is further shown in Table 3 for 10 samples alone. The findings demonstrate that even though switches are distributed throughout several AP clusters (such as Clusters 0, 2, 23, 36, and 56), they are primarily grouped under Agg Cluster 3. This suggests that inter-cluster aggregation unifies switches with comparable normal and anomalous behavioral patterns into a more comprehensive representative cluster. By enhancing behavioral grouping and anomaly localization, this hierarchical mapping facilitates the effective detection and mitigation of flow table overloading attacks. To enable the mitigation of flow table overloading attacks through early anomaly detection, malicious flow isolation, dynamic flow-rule filtering, rate limiting, adaptive flow entry timeout control, and load redistribution mechanisms that prevent flow table exhaustion and improve SDN resilience, inter-cluster analysis was subsequently carried out to further separate normal and anomalous traffic patterns across clusters.

**Table 3:** Sample switch mapping to agglomerative clusters and affinity propagation.

AP cluster ID	No. of switches
0	5
1	3
2	1
3	9
4	2
……	……
127	4
128	4
129	3
130	2
131	3

Agg cluster ID	No. of switches
0	111
1	7
2	12
3	151
4	19

Sample	Switch ID	AP cluster ID	Agg cluster ID
0	102	0	3
1	270	10	3
2	106	26	3
3	71	16	3
4	188	0	3
5	20	26	3
6	102	26	3
7	121	0	3
8	214	25	3
9	87	26	3
10	99	5	3

In comparison to single-stream models, the clustered input is analyzed through dual streams that extract temporal and spatial features, boosting feature separability by roughly 15–20%. The spatial stream is made up of dense layers (64, 32) with ReLU activation. Both short-term and long-term dependency structures are made possible by the temporal stream’s use of a SimpleRNN (32 units, return sequences) and an LSTM (32 units); the comparatively smaller unit size minimizes computing overhead while retaining adequate sequence representation capacity. Gaussian sampling and regularization using KL divergence provide generalization and robustness to traffic variations due to reconstruction on a 32 dimensional latent space using variational layers (*z*_mean, *z*_log_var). The reconstruction step is crucial when utilizing a VAE to connect and encode these features. Normal traffic is reconstructed with relatively low and constant error values (0.1–0.7), but anomalous or low-volume attack traffic consistently produces greater reconstruction errors (0.75–1). Anomaly sensitivity during simulation is improved by this distinct separation. In a layered architecture, the DCLSTM obtains clustered and abnormally filtered traffic from earlier stages and functions as the last classification module. To identify spatiotemporal correlations, a Conv1D–LSTM classifier subsequently processes the encoded features. Specific temporal patterns are extracted using two Conv1D layers (64 filters, kernel size = 3, max-norm = 3), while weight magnitudes are restricted for better generalization. Batch normalization stabilizes gradient flow, while LeakyReLU activation prevents dead neuron issues. Long-range dependencies are represented by a subsequent LSTM layer (100 units, L2 regularization *λ* = 0.01), where the larger unit size improves sequence learning ability. Prior to the final classification using softmax, fully connected layers (100 units) improve the representation of features. The model is optimized by utilizing Adam (learning rate = 10^−4^), where categorical cross-entropy is used for multi-class differentiation and the lower learning rate guarantees stable upgrades in deep sequential systems with batch size 32.

This design uses the ICWO algorithm as a global optimizer to adjust important hyperparameters of the DDST-IV and DCLSTM classifier. ICWO optimizes latent dimension size, learning rate (0.001), and reconstruction loss weighting for the InfoVarCoder, Conv1D filters (32–128), kernel size, batch size (32), and dropout rate (0.2–0.5), all tuned for the DCLSTM. The proposed ICWO algorithm directly interacts with the deep neural network during the training phase by optimizing the weights and biases of the DDST-IV and Dense CLSTM layers. The optimizer performs adaptive parameter tuning using chaotic search behavior and dynamic position updating mechanisms to improve convergence stability, reduce local optima trapping, and enhance classification accuracy during attack detection. Furthermore, the proposed model uses an end-to-end training strategy, where all modules, including spatial–temporal feature extraction, variational encoding, sequential classification, and optimization components, are trained jointly within a unified architecture. This learning approach allows for efficient propagation of gradients throughout the framework, enhancing the ability of the system in feature learning, parameter optimization, convergence and performance improvement in 5G-SDN networks. A population size of 10 ensures sufficient diversity while maintaining effectiveness in computation. A maximum number of 50 iterations allows for trade-off between convergence and optimality of solutions, whereas a chaos factor of 0.5 allows for adding stochasticity in an attempt to avoid getting trapped in local optima. Early stopping is made possible when loss improvements reach a steady state according to the adjustable convergence threshold. Throughout the search phase, ICWO’s learning rate of 0.001 guarantees consistent variable upgrades.

ICWO increases population diversity and search flexibility by combining chaotic search methods with walrus-inspired exploration and exploitation strategies. The chaotic initialization enhances the dispersion of solutions over the search space, while the adaptive search behavior helps avoid local optima and premature convergence compared to the existing optimization. The fitness function improves overall performance and accelerates convergence (20–25%) by combining reconstruction error and classification accuracy. Here, the hyperparametric description of clustering and the modelled optimizer with layers are given in Tables 4–7. The ICWO optimizer in Table 7 refines the model by dynamically updating solutions, incorporating a chaos factor for adaptive learning, and ensuring efficient convergence.

**Table 4:** Description of clustering details in SDN data plane layer.

Clustering method	Number of clusters	Cluster distribution
Affinity propagation (AP)	Auto-determined	{Cluster 0: X switches, Cluster 1: Y switches, Cluster 2: Z switches, …}
Agglomerative clustering	5	{Cluster 0: A switches, Cluster 1: B switches, Cluster 2: C switches, Cluster 3: D switches, …}

**Table 5:** Parameters of DDST-IV Layers

Hyperparameter	Value
Spatial stream input shape	(Number of spatial features,)
Temporal stream input shape	(Number of temporal features, 1)
Spatial stream layers	Dense (64, ReLU), Dense (32, ReLU)
Temporal stream layers	SimpleRNN (32, ReLU, return_sequences = True), LSTM (32, ReLU), Dense (32, ReLU)
Concatenation layer	Merge spatial and temporal outputs
Variational information encoder	Dense (32) (*z*_mean), Dense (32) (*z*_log_var)
Optimizer	Adam $\left(\beta 1=0.9,\beta 2=0.999,\varepsilon =1e^{-8}\right)$
Loss function	RMSE
Batch size	32
Epochs	25
Validation split	20%
Dropout rate	0.5
Early stopping	Patience = 10
Weight initialization	Xavier/Glorot Uniform
Optimizer	Adam $\left(\beta 1=0.9,\beta 2=0.999,\varepsilon =1e^{-8}\right)$
Latent space dimensionality	32
Sampling distribution	Gaussian
KL divergence regularization	Implicitly handled through reparametrization

**Table 6:** Parameters of dense CLSTM modelled layers.

Parameter	Value
Model layers	Conv1D (64 filters, kernel_size = 3, padding = ‘same’, max_norm = 3)
	LeakyReLU Activation
	BatchNormalization
	Dropout (0.5)
	Conv1D (64 filters, kernel_size = 3, padding = ‘same’, max_norm = 3)
	LeakyReLU Activation
	BatchNormalization
	Dropout (0.5)
	LSTM (100 units, kernel_regularizer = l2(0.01), return_sequences = False, max_norm = 3)
	Dropout (0.5)
	Dense (100 units, kernel_regularizer = l2(0.01), max_norm = 3)
	LeakyReLU Activation
	Dropout (0.5)
	Dense (num_classes, activation = ‘softmax’)
Optimizer	Adam
Learning rate	0.0001
Loss function	Categorical cross-entropy
Epochs	25
Batch size	32

**Table 7:** Parameters of ICWO optimizer

ICWO optimizer details	
Parameter	Value
Number of solutions	10 (population size)
Convergence threshold	Dynamic (based on best loss improvement)
Chaos factor	0.5 (controls perturbation level)
Learning rate	0.001
Max iterations	50 (adjustable for convergence)
Best solution	Dynamically updated based on loss improvement

## 5 Results and performance evaluation

**Figure 7:**
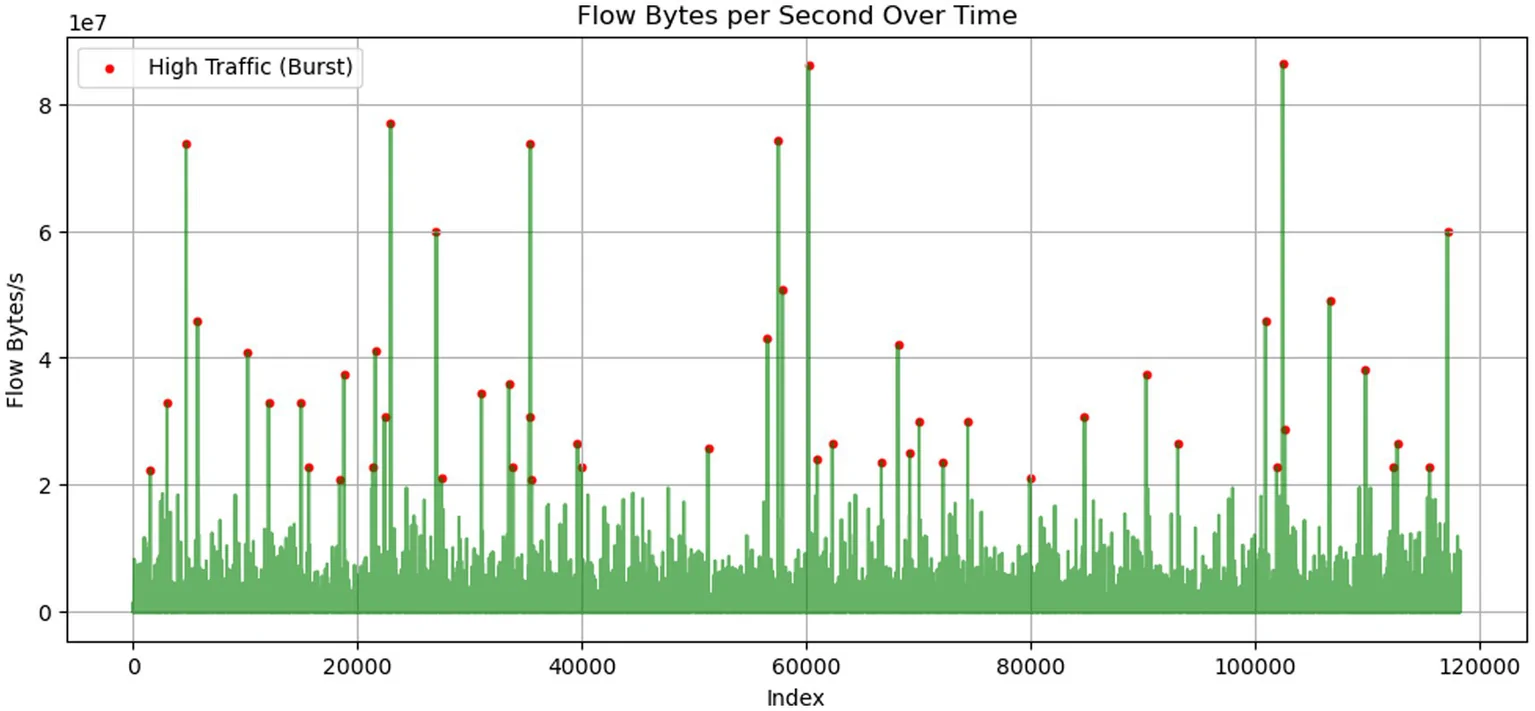
Performance of flow bytes over time (5G SDN dataset).

The following section shows the simulation result of the proposed model on the 5G-SDN network. In order to fully examine the efficiency of the system in detecting and responding to SDN security threats, the following evaluation metrics are considered: accuracy, recall, precision, F1-score, specificity, MAE, RMSE, detection time, throughput and computational cost. The results demonstrate efficient computation and improved security detection capabilities. The variation in Flow Bytes per Second throughout the 5G-SDN dataset is displayed in Figure 7. The sample index (0–120,000) is represented by the x-axis, while the traffic rate is indicated by the y-axis. Normal traffic is indicated by most values staying below 2 × 10^7^ bytes/s (20 MB/s). But there are noticeable bursts that exceed 8–9 × 10^7^ bytes/s (80–90 MB/s). These red-highlighted high bursts differ from typical patterns and may be indicative of abnormal or attack-related behavior, including DoS or Brute force. These patterns indicate structured data transmission and are essential for developing algorithms that optimize data flow by predicting changes and enabling real-time network adjustments.

**Figure 8:**
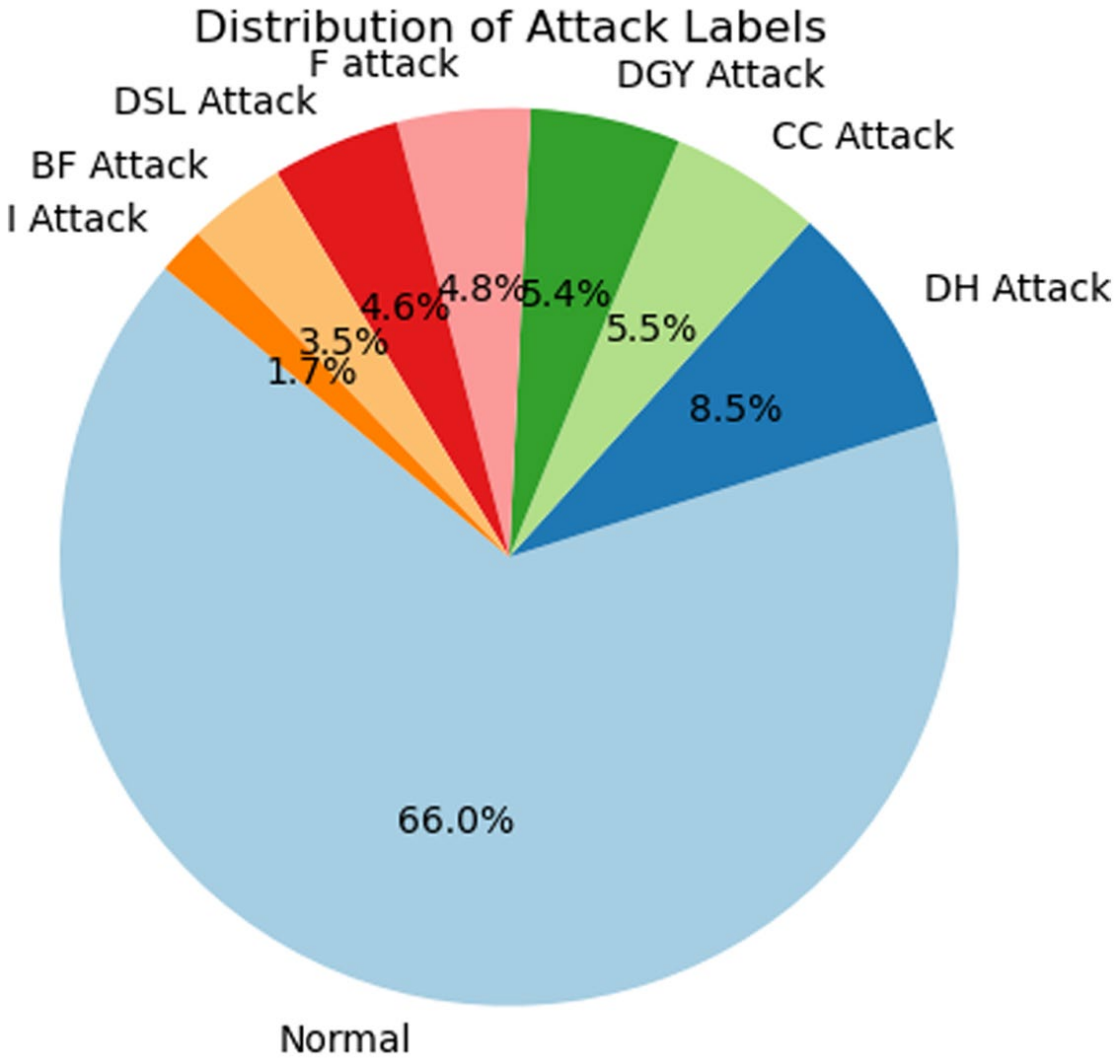
Distribution of 5G SDN dataset.

**Figure 9:**
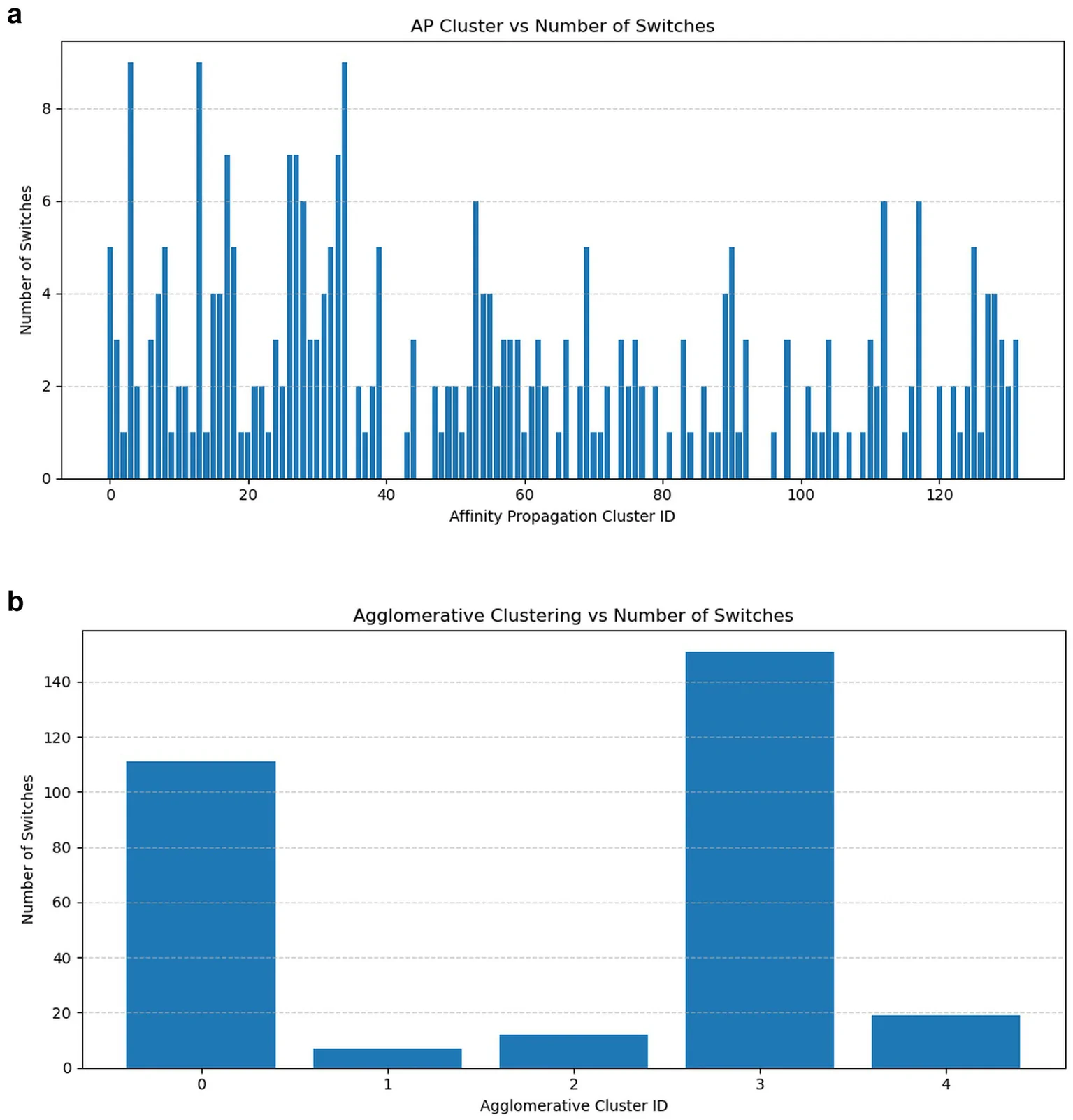
**(a)** Affinity propagation cluster sizes and **(b)** agglomerative cluster analysis.

Figure 8 illustrates the range of normal and Attack variations in labels with percentages. Figure 9 shows the variations of cluster sizes, with the x-axis representing Cluster IDs and the y-axis showing the number of switches per cluster. The varying heights of the bars indicate significant differences in cluster sizes, revealing a wide range of switch distributions.

This variation in cluster sizes is critical for training predictive models, as shown in Figure 9a, where 131 clusters were formed. To prevent having too many or too few clusters, the preference value (−100) was set to the median similarity, and stability is ensured by the damping factor (0.9). It has been proven that the algorithm works efficiently under different traffic scenarios and parameter values. The large clusters comprising many samples provide good data sets that help in predicting regular attacks, while the small clusters identify rare attacks that are significant although they are not regular enough. As can be seen from Figure 9b, Cluster 4, which contains nearly 20 switches indicates a significant behavior of the network. On the other hand, Clusters 1 and 2 which are the two smallest clusters containing just more than the minimum number of switches, indicate irregular or uncommon behaviors. These types of clusters need to be identified because they may represent the uncommon attacks. This research highlights the significance of analyzing the cluster distribution with respect to network security, as the network administrators need to concentrate on Clusters 0 and 3.

To test the stability of the exemplars and clusters across the different iterations, convergence tests have been carried out to test the stability of the APCHC clustering algorithm. Empirical results reveal that there is convergence within a certain number of iterations for different traffic loads. The various measurements of similarities, like the cosine similarity measure, have also been considered for sensitivity analysis and show similar clustering behavior with less variations (<3%). To assess scalability, the number of switches was expanded from small-scale to large-scale deployments (nearly 2000 switches). This confirmed near-linear computational increase and robust clustering behavior even in situations when flow table overloading persisted.

**Figure 10:**
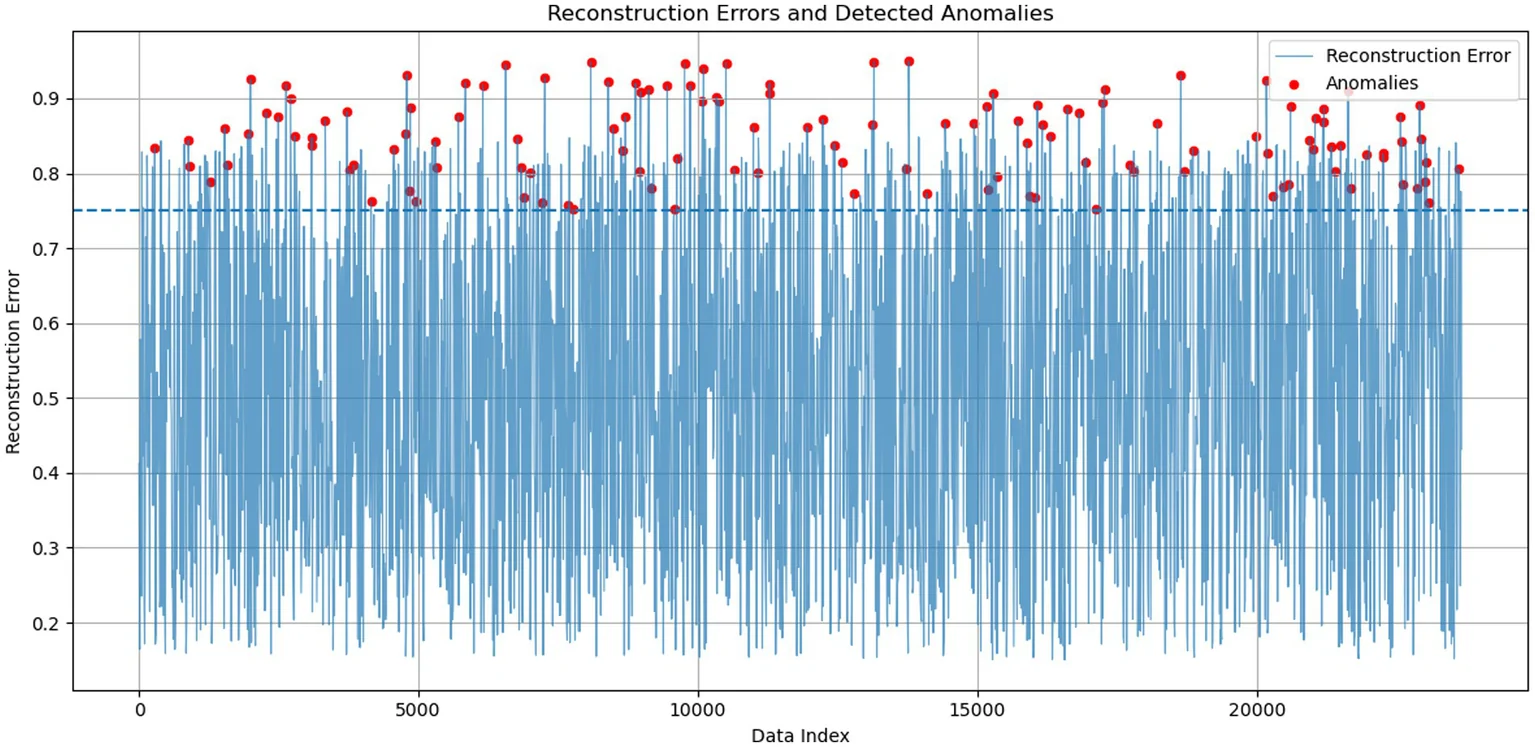
Reconstruction errors vs. data index of detected anomalies.

At the same time, Figure 10 displays the reconstruction errors and detected anomalies of the predictive model. The x-axis represents data indices from 0 to 23,670, while the y-axis shows reconstruction errors from 0.1 to 0.9. Blue vertical lines indicate the reconstruction errors, and red dots represent detected anomalies. The model effectively identifies these anomalies, which are essential for predicting potential attacks in a 5G-SDN network.

The performance evaluation of the suggested model of Attack Prediction Affinity Cohesive Clustering and Dense ICWO-CLSTM with Dual Info-Varcoder in a 5G-SDN network for different attacks, with unknown attacks, is shown in Tables 8, 9.

**Table 8:** Performance of the proposed algorithm for multi-class attacks using 5G SDN dataset.

Attack types	No. of epochs	Multi-classification attacks using 5G SDN dataset for the proposed model								
		Accuracy	Precision	Recall	F1-score	Specificity	Training time (s)	Detection time (s)	MAE	RMSE
Cross-cascaded attacks	5	0.623	0.623	0.634	0.621	0.645	85.67	3.68	0.4123	0.7543
	10	0.741	0.734	0.767	0.734	0.787	143.56	2.95	0.3657	0.6786
	15	0.882	0.897	0.898	0.876	0.899	220.58	2.40	0.2877	0.5242
	20	0.953	0.967	0.956	0.945	0.967	318.05	2.09	0.2432	0.4654
	25	0.983	0.982	0.984	0.985	0.99	498.26	1.74	0.2143	0.3940
Fraggle attacks	5	0.712	0.734	0.743	0.722	0.746	85.67	3.68	0.4112	0.7456
	10	0.920	0.932	0.902	0.912	0.923	143.56	2.95	0.3642	0.6434
	15	0.943	0.943	0.943	0.934	0.943	220.58	2.40	0.2764	0.5125
	20	0.965	0.981	0.976	0.967	0.986	318.05	2.09	0.2321	0.4543
	25	0.985	0.983	0.986	0.985	0.989	498.26	1.74	0.2134	0.3943
DoS Goldeneye attacks	5	0.526	0.532	0.532	0.522	0.545	85.67	3.68	0.4231	0.7678
	10	0.765	0.732	0.734	0.765	0.767	143.56	2.95	0.3765	0.6654
	15	0.812	0.808	0.812	0.812	0.813	220.58	2.40	0.2876	0.5321
	20	0.936	0.943	0.934	0.932	0.945	318.05	2.09	0.2453	0.4765
	25	0.986	0.987	0.988	0.986	0.989	498.26	1.74	0.2201	0.3933
DoS Slow Loris attacks	5	0.621	0.621	0.632	0.650	0.634	85.67	3.68	0.4125	0.7454
	10	0.874	0.878	0.876	0.856	0.876	143.56	2.95	0.3567	0.6543
	15	0.899	0.897	0.91	0.889	0.909	220.58	2.40	0.2812	0.5098
	20	0.964	0.981	0.976	0.966	0.980	318.05	2.09	0.2534	0.4654
	25	0.985	0.982	0.986	0.984	0.991	498.26	1.74	0.2132	0.3922
DoS Hulk attacks	5	0.695	0.687	0.689	0.689	0.698	85.67	3.68	0.4264	0.7765
	10	0.743	0.754	0.767	0.767	0.767	143.56	2.95	0.3654	0.6654
	15	0.912	0.912	0.921	0.912	0.921	220.58	2.40	0.2876	0.5112
	20	0.965	0.975	0.981	0.965	0.987	318.05	2.09	0.2432	0.4655
	25	0.982	0.981	0.987	0.985	0.992	498.26	1.74	0.2109	0.3939
Brute force attacks	5	0.534	0.532	0.545	0.532	0.565	85.67	3.68	0.4354	0.7998
	10	0.628	0.687	0.694	0.689	0.709	143.56	2.95	0.3854	0.6654
	15	0.853	0.854	0.865	0.845	0.865	220.58	2.40	0.2876	0.5342
	20	0.94	0.967	0.943	0.943	0.945	318.05	2.09	0.2564	0.4543
	25	0.986	0.986	0.988	0.987	0.989	498.26	1.74	0.2123	0.3946
Infiltration attacks	5	0.737	0.754	0.734	0.732	0.766	85.67	3.68	0.4055	0.7446
	10	0.855	0.867	0.865	0.844	0.878	143.56	2.95	0.3543	0.6453
	15	0.965	0.965	0.972	0.965	0.981	220.58	2.40	0.2765	0.5087
	20	0.98	0.976	0.981	0.934	0.990	318.05	2.09	0.2342	0.4654
	25	0.986	0.987	0.989	0.988	0.992	498.26	1.74	0.2100	0.3953

**Table 9:** Performance of unknown attacks.

Attack type: unknown attacks	
Performance metrics	Value
Accuracy	0.982 ± 0.003
Precision	0.979 ± 0.002
Recall	0.981 ± 0.002
F1-score	0.980 ± 0.003
Specificity	0.984 ± 0.002
RMSE	0.7600
MAE	0.4430
Detection time	2.1300 sec

**Figure 11:**
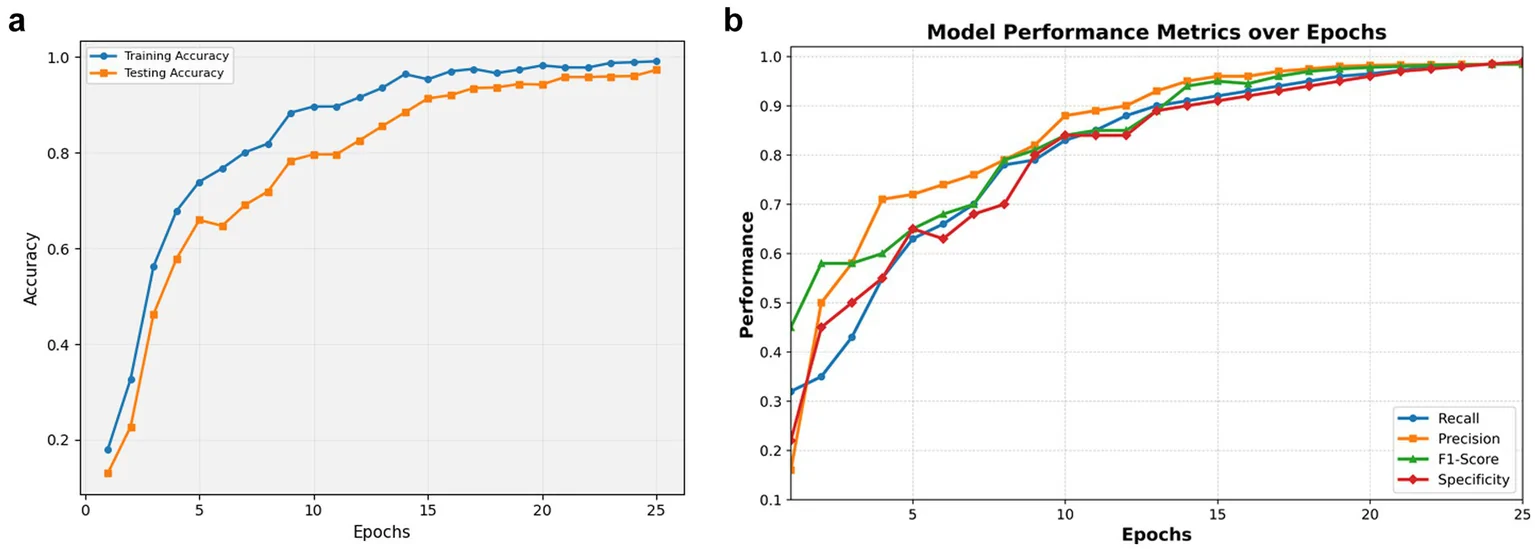
Analysis of **(a)** training with testing accuracy and **(b)** recall, precision, F1-score, and specificity of the proposed model.

Further, Figure 11 illustrates the performance metrics that are used to evaluate the effectiveness of the model to forecast the total number of attack types. Figure 11a shows the training (blue line) and testing (yellow line) accuracy of a model predicting attacks in a 5G-SDN network over 25 epochs. The accuracy rises gradually, reaching 0.96 by epoch 20 and reaching an accuracy of 0.985 ± 0.002 during the 25th epoch. Figure 11b shows the recall, precision, F1-score, and Specificity progression over 25 epochs for the proposed model. The recall approaches 0.986 ± 0.002 at epoch 25, indicating effective detection. During the 25th epoch, the precision equals 0.984 ± 0.003, which indicates the stability of the model in terms of classification after training. During the 25th epoch, the F1-score and Specificity of the model are about 0.985 ± 0.003 and 0.989 ± 0.002, respectively. This means that the efficiency of the model utilizing Affinity Cohesive Clustering, Dual Info-Varcoder, Dense CLSTM, and ICWO is confirmed.

**Figure 12:**
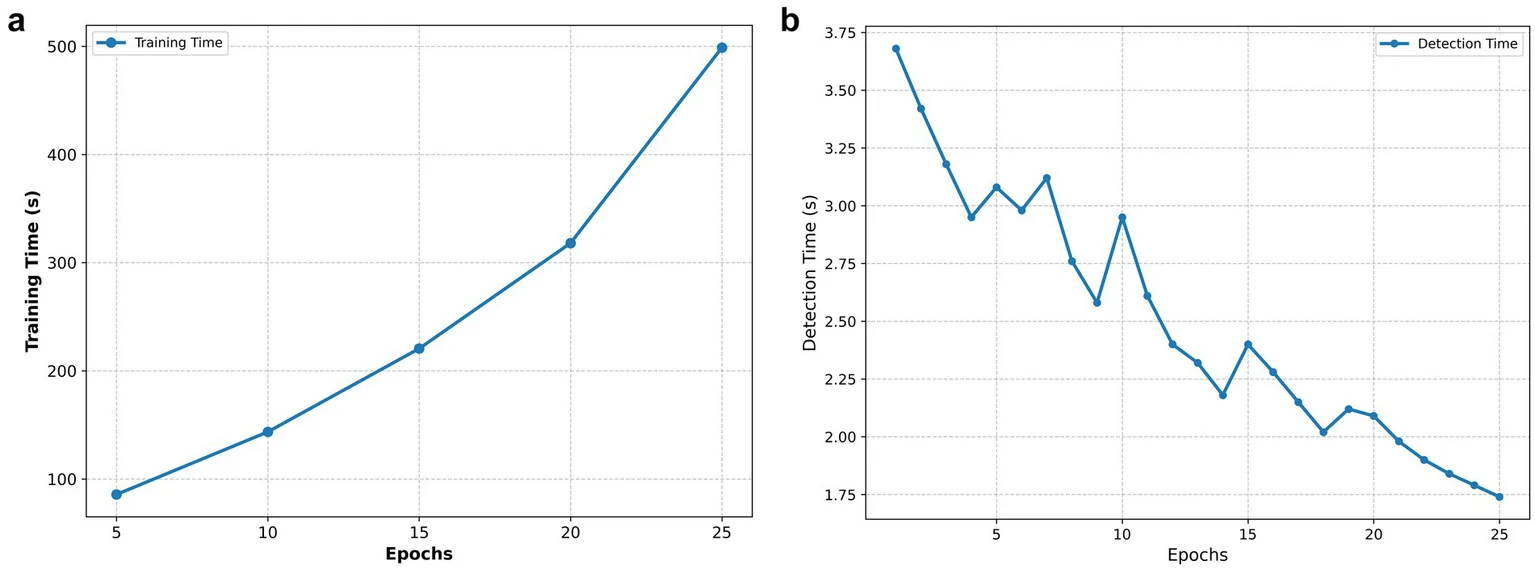
**(a)** Training time and **(b)** SDN detection time analysis of proposed model (5G-SDN dataset).

Figure 12a illustrates how the cumulative training time increases steadily with an increase in the number of epochs. Beginning from 0 s at epoch 0, the training time increases linearly to reach 85.67 s at epoch 5, 143.56 s at epoch 10 and up to 498.26 s at epoch 25. The linear graph indicates the continuous training process without peaks in computational power usage. In Figure 12b, starting from 0 s at epoch 0, the detection time increases continuously to reach 3.68 s at epoch 5, 2.95 s at epoch 10 and 1.74 s at epoch 25. The peaks in detection time could indicate instances where the model comes across complex attack patterns and takes more time to detect them. On the other hand, troughs could indicate cases where the prediction is faster at a shorter detection time.

**Figure 13:**
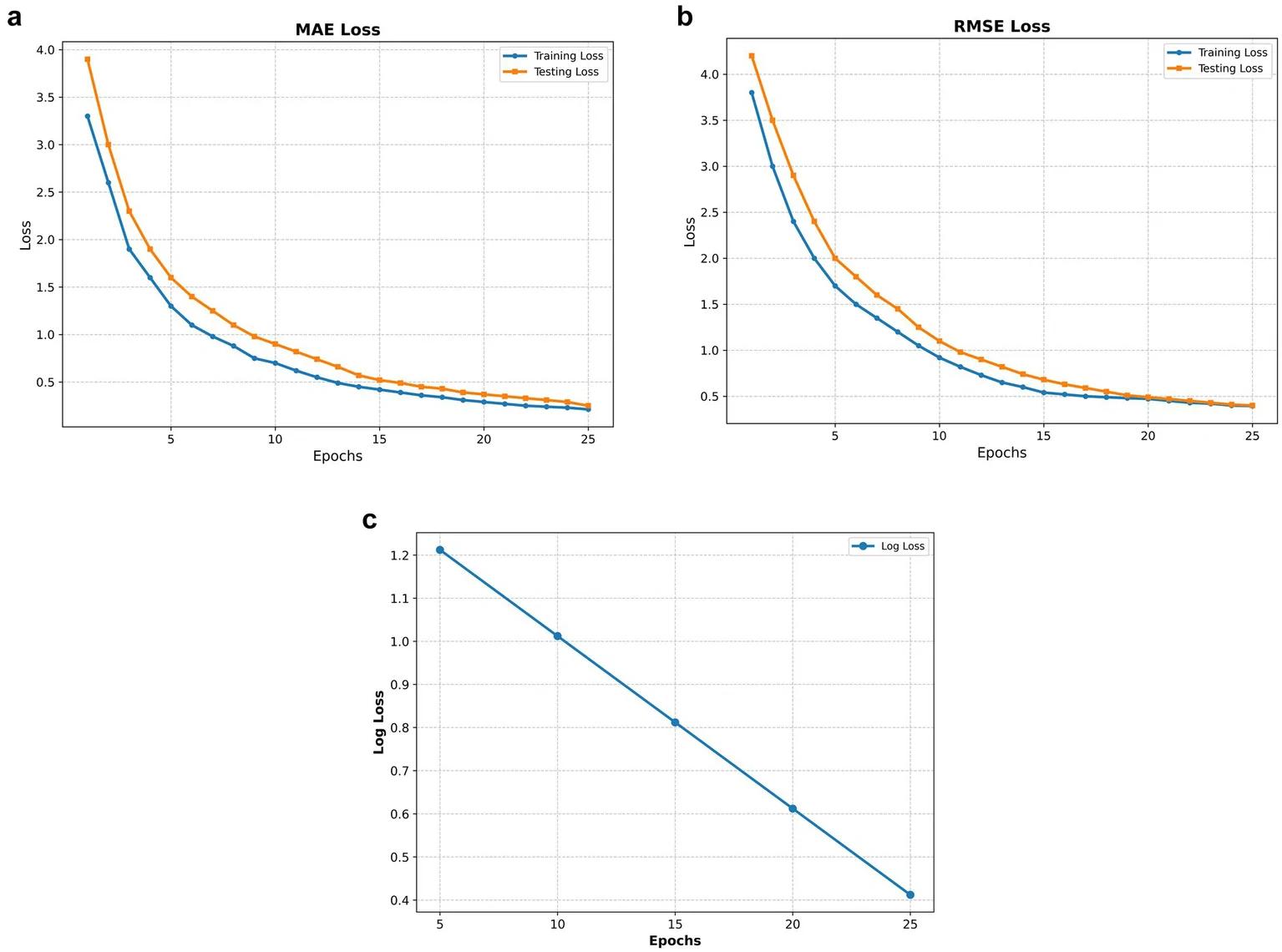
**(a)** MAE **(b)** RMSE, and **(c)** log loss as a function of epoch.

Figures 13a,b show the MAE and RMSE evaluation of the proposed model for 25 epochs. Here, the proposed model gives a minimum MAE of 0.2120 and RMSE of 0.3944 at the 25th epoch and after that, it remains constant. From this, it can be concluded that the proposed model is efficient enough to perform attack prediction on 5G-SDN networks with respect to training data. Figure 13c shows Log Loss of 0.411 at 25 epochs of the model for attack prediction and proposes that the proposed model has a capability to learn attack predictions in a very short period of time. Simulation reveals the throughput of 25 epochs where 3,218 samples/s remain constant, proposing the requirement of continuous monitoring for network efficiency. The sudden decrease and stabilization of CPU utilization values post an increase in CPU utilization values propose the successful mitigation of the attack or any anomaly and hence efficient functioning of the network. The sudden drop of 3.3% at the 25th epoch reveals the quick utilization of the CPU by the model. This highlights the effectiveness of Affinity Cohesive Clustering for attack prediction and management in a 5G-SDN network.

**Figure 14:**
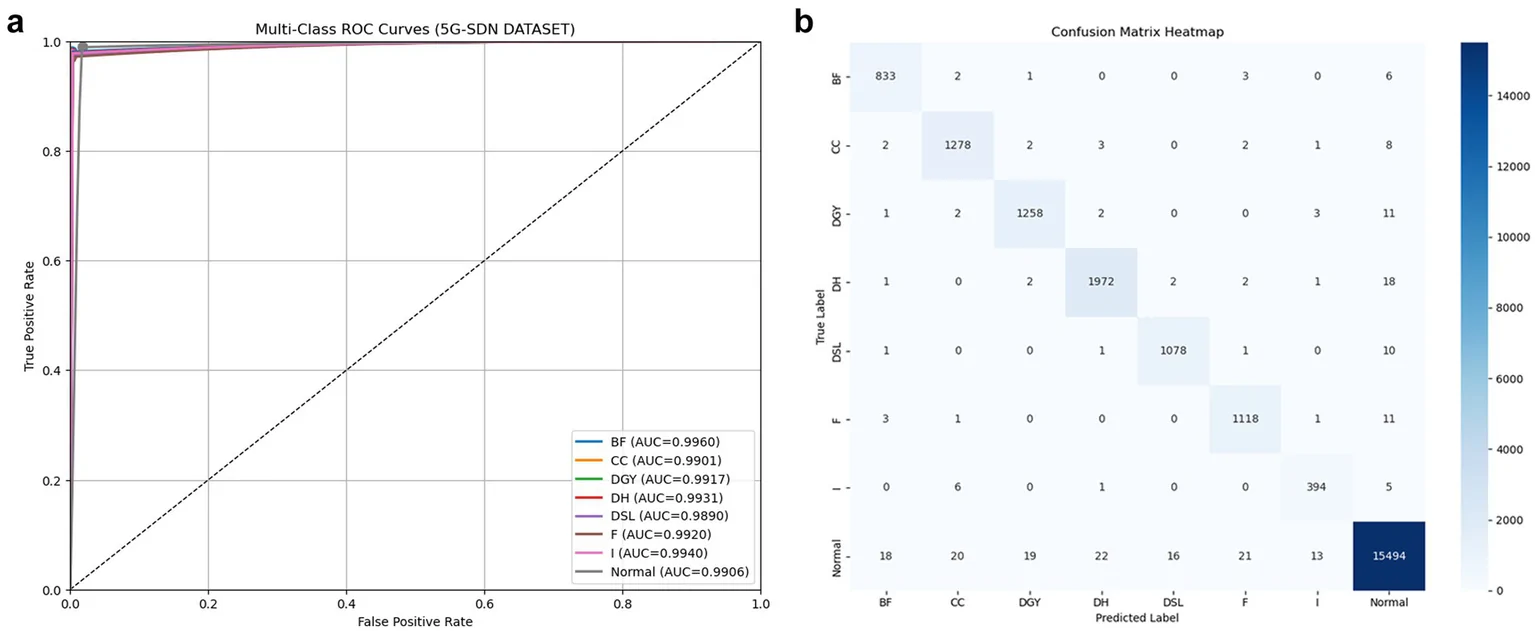
Analysis of **(a)** ROC curve and **(b)** confusion matrix on 5G-SDN dataset.

The Receiver Operating Characteristic (ROC) curve for the anticipated model is shown in Figure 14a. The AUC for each class on the ROC curves shows that the model achieves better classification for all classes. Strong generalization and class discrimination, rather than memorization are proposed by the consistently high AUC values across all attack categories in this study, where the ROC curves are derived from the independent test set. High AUC values by themselves do not indicate overfitting; additionally, statistical analysis and validation-based hyperparameter adjustment were used to reduce overfitting and confirm the robustness of the model. In Figure 14b, a confusion matrix determines the prediction of different attacks. Out of 23,670 test records, the diagonal values indicate correct predictions for each attack type, with high numbers: 833 for BF Attack, 1,278 for CC Attack, 1,258 for DGY Attack, 1972 for DH Attack, 1,078 for DSL Attack, 1,118 for F Attack, 394 for Infiltration Attack. The large figures along the diagonal show that the classifier has done a great job in correctly categorizing majority of the attacks. Due to the negligible off-diagonal misclassifications in the confusion matrix, we can conclude that most attack instances have been correctly classified. Because only a small number of normal traffic instances have been wrongly classified as attacks, the FPR is very low. In the same way, the low off-diagonal numbers suggest a low false negative rate, which means that attack traffic is infrequently mistaken for normal traffic. The framework’s capacity to learn discriminative spatial–temporal indications and successfully distinguish intricate attack patterns is responsible for this performance improvement, which leads to enhanced attack detection and decreased misclassification.

### 5.1 Comparative evaluation of the proposed model with existing approaches

**Figure 15:**
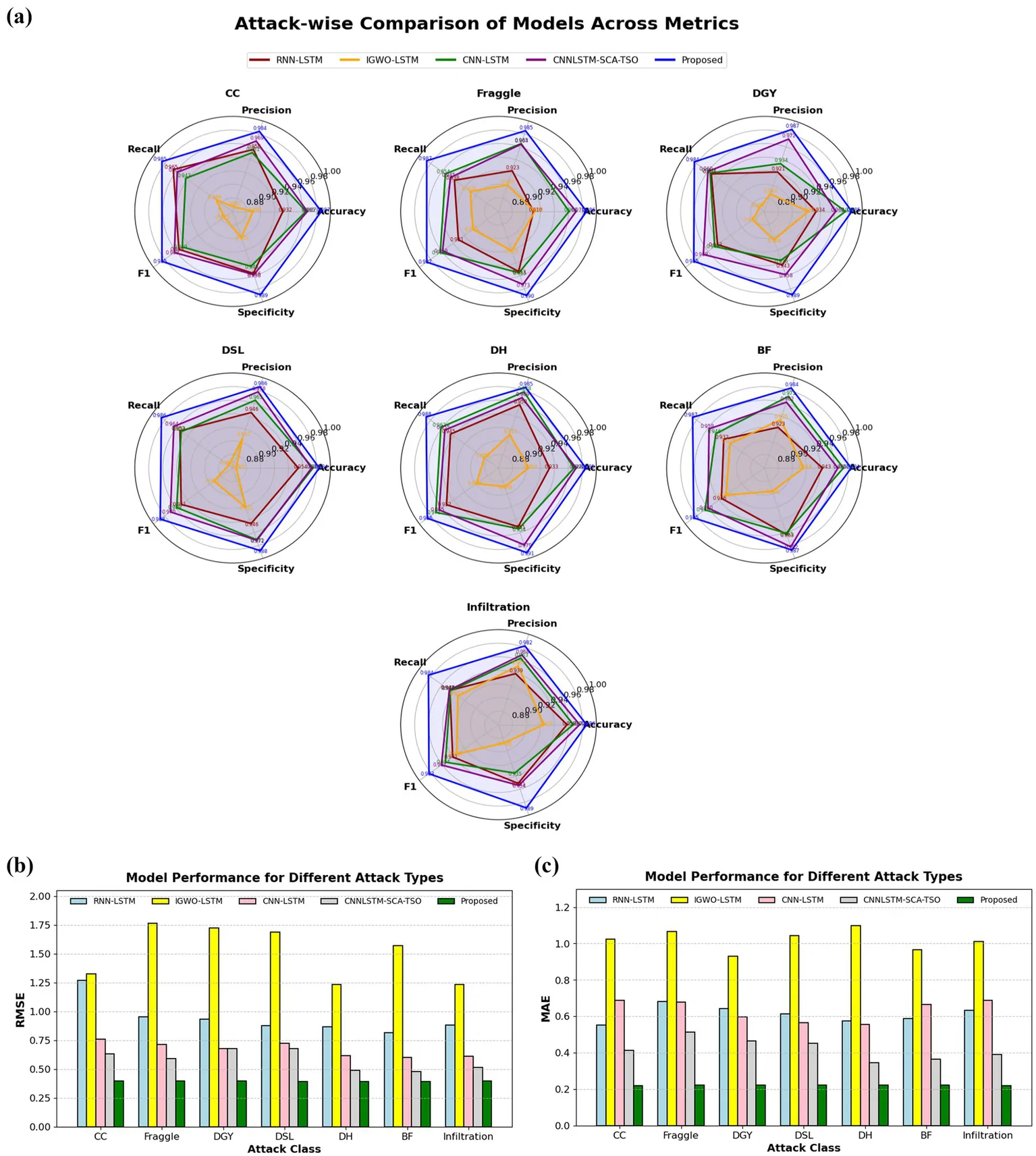
Assessment of **(a)** accuracy, precision, recall, F1-score and specificity, **(b)** RMSE and **(c)** MAE across different types of attacks of various models.

In this section, we present the comparison of different DL models along with the Proposed Dense CLSTM with Dual Info-Varcoder and Attack Prediction Affinity Cohesive Clustering on a 5G-SDN network, wherein the performance of the proposed model is compared with that of other DL models in Tables 10, 11. Table 10 shows the different classification metrics for different categorization of attacks. The DL models are RNN-LSTM (Alshra’a et al., 2021), IGWO-LSTM (Liu et al., 2024), CNN-LSTM (Afroj et al., 2024) and CNNLSTM-SCA-TSO (Jaber Alhilo and Koyuncu, 2024). Figure 15 compares the (a) accuracy, precision, recall, F1-scores, and specificity, (b) RMSE and (c) MAE of different DL models versus the proposed model across various attack classes.

**Table 10:** Comparative analysis of DL models and proposed approach using 5G-SDN dataset.

Classification metrics	Attack types	DL Models for different classification metrics using 5G SDN dataset						
		RNN-LSTM (Alshra’a et al., 2021)	IGWO-LSTM (Liu et al., 2024)	CNN-LSTM (Afroj et al., 2024)	CNNLSTM with SCA-TSO (Jaber Alhilo and Koyuncu, 2024)	APCHC -DDST-IV-LSTM	APCHC -DDST-IV-DC-CLSTM	APCHC-DDST-IV-DC-CLSTM-ICWO (proposed work)
Accuracy	Cross-cascaded attacks	0.932	0.889	0.965	0.967	0.901	0.951	0.987
	Fraggle attacks	0.910	0.912	0.961	0.971	0.898	0.942	0.986
	DoS Goldeneye attacks	0.934	0.923	0.976	0.962	0.932	0.966	0.985
	DoS Slow Loris attacks	0.954	0.865	0.975	0.973	0.956	0.967	0.984
	DoS Hulk attacks	0.933	0.903	0.968	0.971	0.945	0.954	0.985
	Brute force attacks	0.943	0.916	0.973	0.965	0.936	0.953	0.984
	Infiltration attacks	0.958	0.925	0.967	0.976	0.943	0.964	0.986
Precision	Cross-cascaded attacks	0.956	0.867	0.951	0.969	0.901	0.957	0.984
	Fraggle attacks	0.923	0.901	0.965	0.964	0.912	0.943	0.985
	DoS Goldeneye attacks	0.921	0.887	0.934	0.972	0.923	0.956	0.987
	DoS Slow Loris attacks	0.946	0.907	0.965	0.978	0.924	0.969	0.986
	DoS Hulk attacks	0.958	0.912	0.978	0.969	0.928	0.951	0.985
	Brute force attacks	0.923	0.935	0.971	0.962	0.938	0.968	0.984
	Infiltration attacks	0.939	0.951	0.963	0.968	0.935	0.970	0.982
Recall	Cross-cascaded attacks	0.965	0.889	0.943	0.958	0.912	0.965	0.985
	Fraggle attacks	0.938	0.910	0.954	0.945	0.905	0.959	0.987
	DoS Goldeneye attacks	0.954	0.869	0.956	0.960	0.915	0.962	0.984
	DoS Slow Loris attacks	0.953	0.867	0.951	0.964	0.899	0.953	0.986
	DoS Hulk attacks	0.945	0.885	0.962	0.955	0.883	0.966	0.988
	Brute force attacks	0.932	0.921	0.946	0.958	0.941	0.971	0.987
	Infiltration attacks	0.946	0.932	0.945	0.947	0.943	0.965	0.984
F1 score	Cross-cascaded attacks	0.955	0.875	0.949	0.963	0.905	0.962	0.985
	Fraggle attacks	0.931	0.905	0.963	0.959	0.908	0.956	0.987
	DoS Goldeneye attacks	0.943	0.881	0.948	0.968	0.919	0.960	0.985
	DoS Slow Loris attacks	0.951	0.893	0.959	0.970	0.919	0.966	0.988
	DoS Hulk attacks	0.952	0.899	0.971	0.965	0.904	0.964	0.986
	Brute force attacks	0.936	0.928	0.965	0.960	0.940	0.970	0.985
	Infiltration attacks	0.941	0.934	0.954	0.961	0.939	0.969	0.983
Specificity	Cross-cascaded attacks	0.956	0.901	0.945	0.958	0.920	0.964	0.989
	Fraggle attacks	0.953	0.921	0.955	0.973	0.921	0.974	0.990
	DoS Goldeneye attacks	0.943	0.904	0.936	0.958	0.932	0.970	0.989
	DoS Slow Loris attacks	0.946	0.921	0.971	0.972	0.926	0.976	0.988
	DoS Hulk attacks	0.951	0.889	0.954	0.979	0.909	0.969	0.991
	Brute force attacks	0.963	0.896	0.961	0.982	0.932	0.961	0.987
	Infiltration attacks	0.951	0.888	0.935	0.954	0.927	0.969	0.989
RMSE	Cross-cascaded attacks	1.268	1.327	0.756	0.632	1.023	0.643	0.3969
	Fraggle attacks	0.951	1.765	0.712	0.590	0.912	0.654	0.3951
	DoS Goldeneye attacks	0.934	1.723	0.678	0.678	1.032	0.667	0.3947
	DoS Slow Loris attacks	0.876	1.687	0.722	0.676	1.064	0.654	0.3937
	DoS Hulk attacks	0.867	1.234	0.614	0.489	1.131	0.612	0.3921
	Brute force attacks	0.812	1.567	0.598	0.476	1.053	0.522	0.3926
	Infiltration attacks	0.879	1.231	0.612	0.514	1.154	0.599	0.3950
MAE	Cross-cascaded attacks	0.553	1.024	0.689	0.412	0.910	0.346	0.2200
	Fraggle attacks	0.683	1.067	0.678	0.512	0.853	0.564	0.2210
	DoS Goldeneye attacks	0.643	0.931	0.597	0.465	0.964	0.443	0.2224
	DoS Slow Loris attacks	0.612	1.043	0.564	0.453	0.923	0.412	0.2236
	DoS Hulk attacks	0.576	1.098	0.554	0.345	0.909	0.353	0.2220
	Brute force attacks	0.587	0.965	0.665	0.365	0.765	0.354	0.2227
	Infiltration attacks	0.632	1.011	0.689	0.390	0.731	0.343	0.2206
Training time (s)	Cross-cascaded attacks	876.34	915.56	743.78	645.89	612.09	532.18	498.04
	Fraggle attacks	876.34	915.56	743.78	645.89	612.09	532.18	498.04
	DoS Goldeneye attacks	876.34	915.56	743.78	645.89	612.09	532.18	498.04
	DoS Slow Loris attacks	876.34	915.56	743.78	645.89	612.09	532.18	498.04
	DoS Hulk attacks	876.34	915.56	743.78	645.89	612.09	532.18	498.04
	Brute force attacks	876.34	915.56	743.78	645.89	612.09	532.18	498.04
	Infiltration attacks	876.34	915.56	743.78	645.89	612.09	532.18	498.04
SDN traffic detection time (s)	Cross-cascaded attacks	2.56	2.79	2.23	2.05	1.98	1.86	1.74
	Fraggle attacks	2.56	2.79	2.23	2.05	1.98	1.86	1.74
	DoS Goldeneye attacks	2.56	2.79	2.23	2.05	1.98	1.86	1.74
	DoS Slow Loris attacks	2.56	2.79	2.23	2.05	1.98	1.86	1.74
	DoS Hulk attacks	2.56	2.79	2.23	2.05	1.98	1.86	1.74
	Brute force attacks	2.56	2.79	2.23	2.05	1.98	1.86	1.74
	Infiltration attacks	2.56	2.79	2.23	2.05	1.98	1.86	1.74

**Table 11:** Comparative analysis of DL models and proposed approach using 5G-SDN dataset.

Evaluation metrics	No. of epochs	DL Models for different evaluation metrics using 5G SDN dataset						
		RNN-LSTM (Alshra’a et al., 2021)	IGWO-LSTM (Liu et al., 2024)	CNN-LSTM (Afroj et al., 2024)	CNNLSTM with SCA-TSO (Jaber Alhilo and Koyuncu, 2024).	APCHC -DDST-IV -LSTM	APCHC -DDST-IV-DC-CLSTM	APCHC-DDST-IV-DC-CLSTM -ICWO (proposed work)
Accuracy	5	0.512	0.414	0.523	0.654	0.513	0.685	0.744
	10	0.698	0.575	0.656	0.765	0.636	0.775	0.876
	15	0.812	0.654	0.789	0.823	0.781	0.902	0.956
	20	0.887	0.739	0.876	0.918	0.834	0.946	0.967
	25	0.943	0.902	0.973	0.965	0.925	0.961	0.985
Throughput (samples/s)	5	678	564	587	689	456	867	1232
	10	778	654	675	786	567	1088	1716
	15	876	698	1010	1231	675	1412	2176
	20	1342	1453	1457	1675	876	1723	2887
	25	1876	1987	2234	2345	1029	2311	3218
CPU utilization (%)	5	11.54	10.62	9.54	9.87	9.99	8.98	8.89
	10	10.66	9.73	8.98	8.65	8.09	7.87	7.33
	15	9.32	8.60	7.65	7.56	7.65	6.67	6.21
	20	8.09	7.43	6.67	6.34	6.78	4.34	4.09
	25	7.45	6.32	5.34	5.25	5.43	3.98	3.32
Log loss	5	1.75	2.16	1.09	0.987	1.321	0.765	0.986
	10	1.19	2.21	1.12	0.976	1.253	0.542	0.654
	15	1.61	1.98	1.23	0.876	0.954	0.482	0.567
	20	1.54	1.94	1.34	0.921	0.564	0.454	0.498
	25	1.43	2.03	1.26	0.879	0.432	0.433	0.411
Latency per sample (ms)	5	2.67	2.45	1.98	2.43	2.65	1.92	1.656
	10	2.88	2.31	1.45	2.16	2.34	1.67	1.324
	15	2.54	1.98	1.23	1.76	1.96	1.06	0.976
	20	1.72	1.65	1.01	1.43	1.45	0.93	0.762
	25	1.32	1.21	0.98	1.03	1.12	0.76	0.465
Energy consumption (J)	5	1.989	1.87	1.76	1.576	1.387	1.272	1.428
	10	1.786	1.76	1.65	1.489	1.286	1.081	1.112
	15	1.676	1.13	1.32	1.358	1.112	0.978	0.897
	20	0.945	0.954	0.987	1.213	0.975	0.834	0.765
	25	0.912	0.939	0.923	0.969	0.898	0.621	0.634

As seen in Figure 15, the proposed approach consistently performs better than the existing DL models on all evaluation metrics. For Fraggle attacks 0.986 and Cross-cascaded attacks 0.987, it attains the most effective accuracy. The model gained precision scores of 0.986 for DoS Goldeneye attacks and 0.987 for DoS Slow Loris. Additionally, it achieves F1-scores of 0.988 for DoS Slow Loris attacks and 0.987 for Fraggle attacks, recording the maximum recall for DoS Hulk attacks of 0.988. Additionally, the model exhibits high specificity, achieving 0.990 for Fraggle attacks and 0.991 for DoS Hulk attacks.

Additionally, it achieves an RMSE of 0.3944 and MAE of 0.2120, demonstrating overall performance by minimizing the average error across all attack types. These metrics are averaged for this category of all types of attacks. As per the findings of the report, the accuracy (0.985 ± 0.002) is constant, which proves the robustness of the model towards different data sets. The precision (0.984 ± 0.003) and recall (0.986 ± 0.002) prove the effectiveness of the model in reducing false positives and false negatives. The F1-score (0.985 ± 0.003) shows the trade-off between precision and recall.

**Figure 16:**
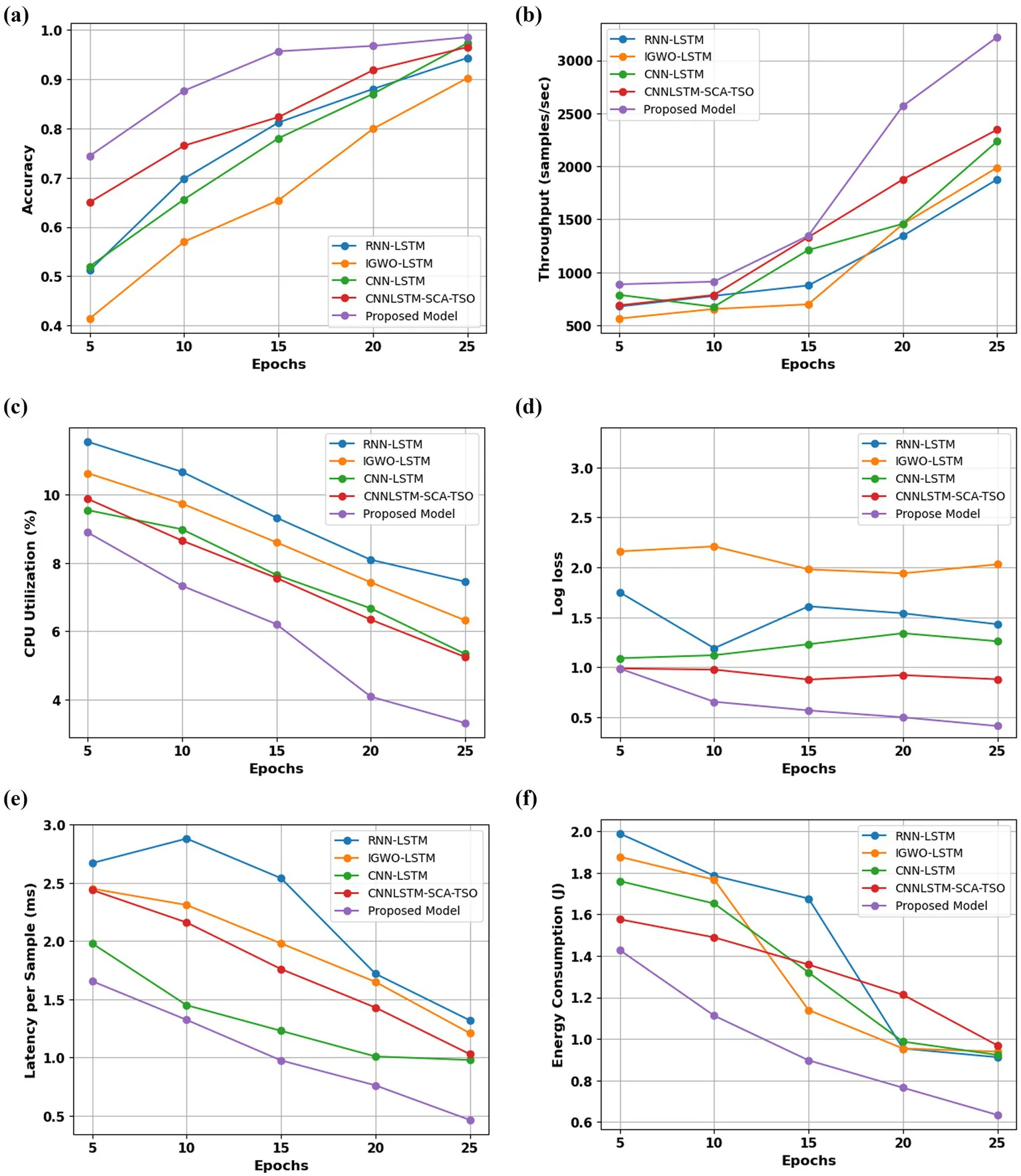
Exploration of **(a)** accuracy, **(b)** throughput, **(c)** CPU utilization, **(d)** log loss rate, **(e)** latency, and **(f)** energy consumption across various epochs.

Table 11 highlights the proposed model’s effectiveness in terms of accuracy (0.985 ± 0.002), high throughput (3,218 samples/s), low CPU Utilization (3.3%), low Log loss (0.411), low latency (0.465), and energy consumption (0.634) over 25 epochs. Hence, this accurate detection of diverse attacks with high precision and efficiency makes it ideal for robust 5G SDN network security. Figure 16 compares the Accuracy, Throughput, CPU Utilization, Log loss, latency per sample (ms) and energy consumption (in Joules) across different models over increasing epochs (5–25).

The proposed model (purple line) outperforms the other deep learning models, including RNN-LSTM (Alshra’a et al., 2021), IGWO-LSTM (Liu et al., 2024), CNN-LSTM (Afroj et al., 2024) and CNNLSTM-SCA-TSO (Jaber Alhilo and Koyuncu, 2024). The performance gap widens at higher epochs, demonstrating the higher efficiency of the proposed approach. Energy consumption by other algorithms varies; for instance, CNNLSTM-SCA-TSO attains its peak near the 20th epoch. This demonstrates that the proposed approach is more efficient in terms of energy consumption.

### 5.2 Statistical significance analysis and model validation

The statistical approach and comparison of baseline models are applied for the validation of the proposed architecture in this section. The use of 5-fold cross-validation and *t*-tests is used in order to prove the validity and reliability of the obtained results. In order to prove the efficacy of the proposed architecture, the comparative analysis is carried out with the help of some popular ML and DL models, which include XGBoost, Transformer, Random Forest and CNN-LSTM models. The analysis is carried out on the basis of such existing criteria as accuracy, precision, recall and F1-score in order to test the effectiveness of every model in the process of detecting complex attacks in 5G-SDN environments.

Table 12 shows the comparative performance analysis among the baseline models and the proposed model. From the results, it is evident that the proposed model performs excellently in classification with an accuracy of 0.985 ± 0.002, precision of 0.984 ± 0.003, and so forth, outperforming all other existing models. In the baseline models, the CNN-LSTM model performs comparatively better owing to its ability to learn spatial and temporal characteristics of traffic data. But the performance of the model is lower compared to the proposed framework owing to its inability to perform advanced clustering, dual-stream feature extraction, and optimization processes as in the APCHC-DDST-IV-CLSTM-ICWO framework. The Transformer model shows excellent performance owing to its ability to learn through the attention mechanism; however, it is less efficient in learning dynamic flow-level SDN traffic patterns compared to the proposed model. Similarly, XGBoost and Random Forest perform poorly in detection due to their inability to learn high-dimensional sequential traffic dependencies and evolving attacks. It is clear from the results that the combination of APCHC clustering, DDST-IV spatial–temporal feature extraction, Dense CLSTM classification, and ICWO optimization improves intrusion detection performance and robustness in a 5G-SDN environment.

**Table 12:** Comparative performance analysis of baseline models and the proposed algorithm.

Model	Accuracy	Precision	Recall	F1-score	Detection time (s)
XGBoost	0.958 ± 0.004	0.953 ± 0.005	0.961 ± 0.004	0.959 ± 0.004	1.562
Transformer	0.965 ± 0.003	0.968 ± 0.004	0.974 ± 0.003	0.970 ± 0.003	2.052
Random Forest	0.942 ± 0.002	0.931 ± 0.002	0.938 ± 0.002	0.936 ± 0.004	1.686
CNN-LSTM	0.961 ± 0.005	0.965 ± 0.003	0.954 ± 0.003	0.963 ± 0.002	2.132
Proposed model	0.985 ± 0.002	0.984 ± 0.003	0.986 ± 0.002	0.985 ± 0.003	1.742

In order to examine the model’s robustness, stability, and ability to generalize, a k-fold cross-validation technique is applied to the 5G-SDN dataset. Cross-validation is a significant evaluation approach that determines the consistency of the model’s performance on various subsets of the dataset while minimizing the risk of overfitting. In this case, the dataset is divided into five folds, where each of them is used for testing one by one, while the rest are used for training purposes. Evaluation of the proposed approach will be conducted based on metrics.

Table 13 shows the cross-validation performance of the proposed framework on five independent folds. The results from experiments clearly show high levels of performance on all five validation folds, which proves the robustness and generalized nature of the proposed intrusion detection framework. The accuracy of the model is observed to have values between 0.985 and 0.988, with precision, recall, and F1-score having corresponding values consistently greater than 0.984, which is an indication of stable classification capability of the model when faced with varied training and testing datasets. The mean accuracy of 0.9854 and the standard deviation of ± 0.0019 show that the model has little variation in performance across different folds, hence proving its good generalization capability. Likewise, the standard deviation values for the precision, recall, and F1-score are low, indicating balanced performance for attacks in both majority and minority classes, which improves the stability, reliability and detection capability in SDN.

**Table 13:** Five-fold cross-validation performance analysis of the proposed model.

Metrics	Method	Fold 1	Fold 2	Fold 3	Fold 4	Fold 5	Mean	Std dev
Accuracy	*Proposed model*	0.985	0.987	0.983	0.984	0.988	0.9854	0.0019
	XGBoost	0.958	0.943	0.938	0.962	0.951	0.9504	0.0091
	Transformer	0.965	0.974	0.970	0.969	0.975	0.9706	0.0037
	Random Forest	0.942	0.956	0.961	0.932	0.948	0.9478	0.0110
	CNN-LSTM	0.961	0.972	0.956	0.966	0.971	0.9652	0.0061
Precision	*Proposed model*	0.984	0.986	0.985	0.983	0.99	0.9856	0.0021
	XGBoost	0.953	0.946	0.940	0.958	0.959	0.9512	0.0077
	Transformer	0.968	0.971	0.965	0.973	0.972	0.9698	0.0029
	Random Forest	0.931	0.951	0.968	0.947	0.954	0.9502	0.0129
	CNN-LSTM	0.965	0.976	0.965	0.973	0.975	0.9708	0.0046
Recall	*Proposed model*	0.986	0.989	0.987	0.986	0.988	0.9872	0.0011
	XGBoost	0.961	0.953	0.958	0.963	0.967	0.9604	0.0049
	Transformer	0.974	0.976	0.972	0.981	0.979	0.9764	0.0033
	Random Forest	0.938	0.946	0.973	0.955	0.961	0.9546	0.0126
	CNN-LSTM	0.954	0.981	0.974	0.979	0.969	0.9714	0.0100
F1-score	*Proposed model*	0.985	0.988	0.989	0.985	0.991	0.9876	0.0023
	XGBoost	0.959	0.950	0.954	0.962	0.966	0.9582	0.0057
	Transformer	0.970	0.975	0.971	0.978	0.977	0.974	0.0030
	Random Forest	0.936	0.951	0.972	0.953	0.958	0.954	0.0121
	CNN-LSTM	0.963	0.980	0.974	0.978	0.973	0.9736	0.0062

In order to test the statistical validity of the performance gain achieved using the proposed model, a paired *t*-test is also performed on the baseline models. The paired *t*-test examines whether the difference in performance achieved using the proposed approach compared to the existing approaches is statistically significant or not. For this purpose, the values of mean difference, *t*-value, *p*-value, and statistical significance level are taken into account.

Table 14 presents the paired *t*-test results comparing the proposed model with XGBoost, Transformer, Random Forest, and CNN-LSTM approaches. The difference is not statistically significant (due to chance) if *p* > 0.05. The difference is statistically significant (probably real) if *p* < 0.05. The results demonstrate that the suggested framework achieves statistically significant gains over all baseline models, with *p*-values less than 0.001 in most cases. The highest mean accuracy improvement of 4.3% is observed against the Random Forest model, indicating the superior capability of the proposed DL architecture in handling complex and high-dimensional 5G-SDN traffic data. Similarly, a substantial improvement of 2.7% is achieved compared with XGBoost, confirming the effectiveness of the proposed spatial–temporal feature extraction and optimization mechanisms. Although the CNN-LSTM gives similar performance, the proposed framework is still better than that one, showing 2.4% improvement in performance with *t*-statistic of 4.859 and a *p*-value of 0.0082. Lower *p*-values and high values of *t*-statistic in all comparisons show that improvements shown by the proposed model are statistically significant and are not the result of random fluctuations in training and testing of the model. Therefore, the statistical analysis supports the validity of the robustness, consistency and effectiveness of the proposed intrusion detection system for the security of 5G-SDN networks against cyber threats. This validation lowers the risk of overfitting or bias and improves the suggested approach’s dependability and generalizability across different data splits.

**Table 14:** Statistical significance test for the suggested model compared to the current models using paired *t*-test.

Model	Mean accuracy difference (%)	*T*-statistic	*p*-Value	Statistical significance
Proposed vs XGBoost	2.7	9.877	0.0005	Significant
Proposed vs Transformer	2.0	7.422	0.0017	Significant
Proposed vs Random Forest	4.3	6.544	0.0028	Significant
Proposed vs CNN-LSTM	2.4	4.859	0.0082	Significant

### 5.3 Computational cost and training time analysis

The computational complexity of an attack detection framework in SDNs running in real-time 5G-SDN setting is crucial for the assessment of the practical implementation possibilities of the attack detection frameworks. Besides the high accuracy of the attack detection, an effective framework should ensure an acceptable level of computation complexity, low latency of the inference process, and faster training process convergence. Thus, the proposed framework will be compared with a few other baseline models on the basis of computational performance measures such as number of parameters, floating point operations per second (FLOPs), training time, and inference time.

Table 15 shows the results for the computational complexity evaluation of various intrusion detection models. From the above results, it can be observed that the proposed model attains a balance between computational overhead and detection accuracy. Despite having a high number of parameters (10.3 million) and the computational cost (5.14 GFLOPs), the model takes less time in both training and inference operations as compared to Transformer and CNN-LSTM models. The Transformer model uses the highest computational resources as it has 12.5 million parameters, 6.85 GFLOPs, a training time of 864 s, and an inference time of 24.7 ms since it performs many attention-based multi-headed computations. Similarly, CNN-LSTM model also utilizes large computational resources due to the convolutional and sequential computations. However, classical ML models like XGBoost and Random Forest require low computational complexity. But their intrusion detection accuracy is relatively poor compared to the proposed model due to their inability to effectively learn complex space–time traffic dependencies. The proposed APCHC-DDST-IV-CLSTM-ICWO model attains a training time of 598 s and an inference latency of 14.1 ms. This achievement is largely owed to the APCHC-based adaptive clustering scheme, effective DDST-IV feature extraction algorithm, optimized Dense CLSTM architecture, and ICWO-based optimization of parameters, which altogether decrease redundant calculations and improve feature learning capacity. The integration of the APCHC, Deep Dual InfoVarCoder, DCLSTM, and ICWO blocks in the proposed system requires much additional computation power, although the growth of detection time remains within an acceptable limit for SDN systems. The significant gains in attack discrimination, temporal dependence modeling, feature representation, and detection precision definitely warrant the additional complexity. Thus, the right balance between the computational expense and detection efficiency is reached by the proposed solution.

**Table 15:** Computational complexity comparison of baseline models and the proposed model.

Model	Params (M)	FLOPs (G)	Training time (s)	Inference time (ms)
XGBoost	1.8 M	0.42	118	8.4
Transformer	12.5 M	6.85	864	24.7
Random Forest	3.2 M	0.73	156	11.9
CNN-LSTM	8.7 M	4.62	612	18.3
Proposed model	10.3 M	5.14	598	14.1

### 5.4 Benchmark dataset-based performance assessment of the proposed model

It is important to evaluate the proposed approach on various data sets in order to show its flexibility in changing conditions of traffic in the network, the type of attacks, and feature distribution. To ensure proper evaluation of the results, the authors have used not only the freely available data sets but also the synthetically created data set for 5G-SDN. Table 16 shows the performance of the proposed method on various data sets in comparison with the created data set of 5G SDN. The following data sets were used [ULAK (Chatzimiltis, 2024), InSDN (Elsayed et al., 2020), CICIDS2017, NSL-KDD]. This test was done to evaluate the possibility of generalizing the results of the proposed framework and its effectiveness in different environments and types of attacks. The performance analysis of the proposed model for different data sets is provided in Table 16. The high average accuracy of the proposed model (0.984, 0.986, and 0.987 for InSDN, CICIDS2017, and NSL-KDD data sets, respectively) shows the effectiveness of the classification performed by the model for all benchmark data sets. Also, the high average F1-score value of 0.986, 0.988, and 0.988 for InSDN, CICIDS2017, and NSL-KDD data sets, respectively, shows the good balance between recall and precision of detection for different types of attacks. The specificity value of the proposed model was above 0.987, which reduces false positives.

**Table 16:** Evaluating proposed model performance across multiple datasets vs. 5G-SDN dataset.

Dataset	Attacks	Performance metrics						
		Accuracy	Precision	Recall	F1-score	Specificity	Train time (s)	Detect time (s)
ULAK (Chatzimiltis, 2024)	DDoS and DoS	0.982	0.980	0.984	0.983	0.985	587.33	2.09
InSDN (Elsayed et al., 2020)	DDoS	0.986	0.985	0.987	0.986	0.989	626.76	2.21
	Probe	0.985	0.988	0.990	0.989	0.991		
	DoS	0.99	0.987	0.989	0.988	0.992		
	BF	0.984	0.985	0.988	0.987	0.988		
	Web Attack	0.986	0.982	0.986	0.984	0.987		
	Botnet	0.972	0.983	0.980	0.982	0.986		
Average		0.984±0.001	0.985±0.002	0.985±0.002	0.986±0.002	0.987±0.003	626.76	2.21
CICIDS-2017 (Canadian Institute for Cybersecurity, 2017)	DDoS	0.989	0.985	0.989	0.988	0.990	582.88	1.97
	DoS	0.988	0.990	0.992	0.991	0.992		
	BF	0.990	0.989	0.991	0.990	0.988		
	Bot	0.988	0.990	0.987	0.989	0.989		
	Web attack	0.984	0.987	0.990	0.988	0.986		
	I attack	0.983	0.985	0.987	0.986	0.985		
Average		0.986±0.002	0.987±0.001	0.989±0.001	0.988±0.001	0.989±0.002	582.88	1.97
NSL-KDD (Tavallaee et al., 2009)	DoS	0.986	0.988	0.991	0.991	0.990	492.45	1.65
	Probe	0.989	0.984	0.986	0.985	0.988		
	U2R	0.990	0.989	0.989	0.991	0.990		
	R2L	0.989	0.987	0.990	0.989	0.993		
Average		0.987±0.001	0.986±0.001	0.989±0.002	0.988±0.002	0.990±0.001	492.45	1.65
Generated (5G-SDN dataset)	CC	0.987	0.983	0.985	0.985	0.989	498.04	1.74
	Fraggle	0.986	0.985	0.987	0.987	0.990		
	DGY	0.985	0.987	0.984	0.985	0.989		
	DSL	0.984	0.986	0.986	0.988	0.988		
	DH	0.985	0.985	0.988	0.986	0.991		
	BF	0.984	0.984	0.987	0.985	0.987		
	I attack	0.986	0.982	0.984	0.983	0.989		
Average		0.985±0.002	0.984±0.003	0.986±0.002	0.985±0.003	0.989±0.002	498.04	1.74

**Figure 17:**
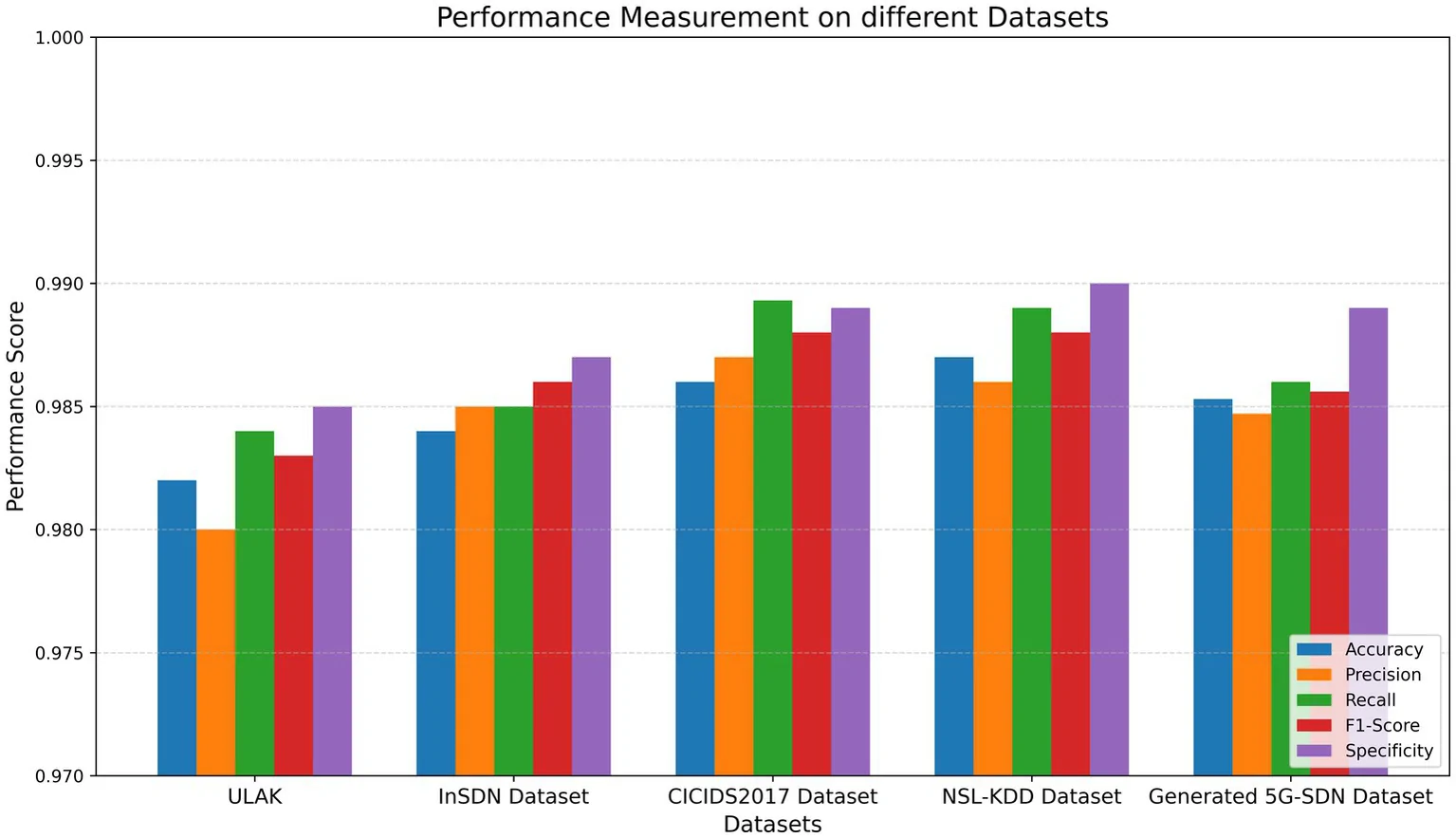
Evaluation of the proposed model on distinct datasets using performance metrics.

Figure 17 presents the performance evaluation of the proposed model with different Benchmark datasets. The ability of the model to detect malicious traffic efficiently for various types of attacks was shown by the NSL-KDD dataset having the highest average accuracy (0.987 ± 0.001) and specificity (0.990 ± 0.001) among benchmark datasets, while the CICIDS2017 dataset exhibited the best average recall (0.989 ± 0.001). Also, the average accuracy of (0.984 ± 0.001) and average F1-score of (0.986 ± 0.002) of the InSDN dataset, consisting of different types of SDN-based attacks like DDoS, Probe, DoS, Brute Force, Web Attack and Botnet attacks, demonstrate competitive results, showing the applicability of the proposed framework for the SDN environment. Moreover, with average accuracy of (0.985 ± 0.002), average recall of (0.986 ± 0.002), average F1-score of (0.985 ± 0.003) and average specificity of (0.989 ± 0.002), the generated 5G-SDN dataset provided comparable performance parameters. Thus, highly efficient detection of complex attack patterns in the dynamically changing SDN environment was obtained. The proposed model does not overfit to the synthetic environment and retains consistent detection capabilities across heterogeneous datasets, as evidenced by the good agreement between the findings achieved on the generated dataset and those seen on publicly accessible benchmark datasets.

The proposed framework demonstrated acceptable training and detection times compared to all datasets in terms of computing efficiency. Due to the greater number of attack categories, InSDN had the longest training time (626.76 s), while its detection time was only 2.21 s. The generated 5G-SDN dataset needed 1.74 s for attack detection, but the NSL-KDD dataset reached a minimum detection time of 1.65 s. These findings demonstrate that the proposed approach may provide precise and effective detection while preserving resilience between generated and benchmark datasets. Overall, the experimental results show that the proposed approach successfully generalizes across several benchmark datasets and a generated 5G-SDN dataset, resolving dataset dependency issues and enhancing the proposed intrusion detection framework’s effectiveness.

**Figure 18:**
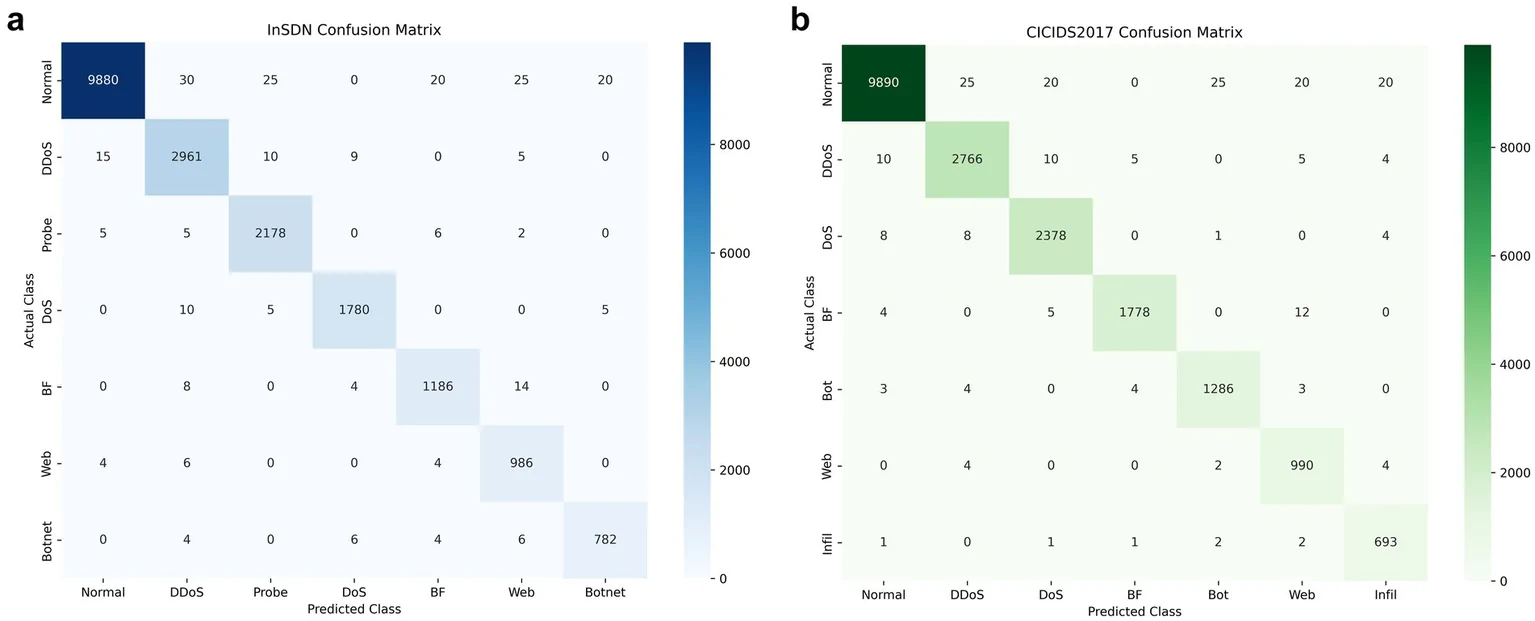
Multiclass confusion matrix of the proposed model **(a)** InSDN dataset **(b)** CICIDS2017 dataset.

This proposed methodology provides a higher accuracy in classification for all classes, as observed in the confusion matrices as shown in Figure 18 (a) InSDN (b) CICIDS2017. Most of the observations are placed on the diagonals, which clearly prove the successful classification of attacks and non-attacks. The model’s excellent discriminative capacity and efficacy in identifying various intrusion types are confirmed by the very small number of off-diagonal entries, which show little confusion among attack categories and are deviated from the actual class and sustain the low FPR. Also, the model performed well across multiple tests and validations on the benchmark datasets. This surpasses the models and datasets in training and detection time, proving its efficiency for real-time applications. The consistently high performance across all datasets validates the effectiveness of the APCHC clustering mechanism, DDST-IV spatial–temporal feature extraction, CLSTM-based attack classification, and ICWO-driven parameter optimization in improving the attack detection capability of 5G-SDN security systems.

### 5.5 Ablation-based performance analysis

According to the observed performance, traditional CNN-LSTM models and other Machine Learning models struggle to accurately define complex attack behaviors that appear across numerous network states and traffic distributions in real 5G, instead learning limited spatial and temporal correlations. On the other hand, in order to better capture intricate spatial–temporal correlations and hidden attack patterns, the proposed approach integrates Affinity Propagation Hierarchical Clustering (APCHC), a Deep Dual-Stream Info-Varcoder, and a Densely Connected ConvLSTM optimized using ICWO. Even while the proposed approach adds more computational complexity, each component helps to improve feature representation, optimize learning, and identify more complex and dynamic cyberattacks in 5G-SDN, all of which lead to better overall performance.

To examine the role played by each component in the proposed model, a module ablation-based performance analysis is conducted by systematically removing or replacing individual modules and examining their impact on the overall intrusion detection performance. The ablation study helps to verify the efficacy of the suggested modules in enhancing attack detection accuracy, feature representation, classification robustness, and optimization efficiency. In addition, the analysis of optimizer replacement demonstrates the efficiency of the proposed ICWO optimizer in comparison to other existing optimizers.

Table 17 shows the ablation research for the proposed approach with the removal of different modules from the framework to show how much each component affects the performance. It is evident from the results that the overall proposed framework performs best when used in full form, having an accuracy of (0.985 ± 0.002), precision of (0.984 ± 0.003), recall of (0.986 ± 0.002), and F1 score of (0.985 ± 0.003). In the case of removal of the APCHC module from the framework, the accuracy decreases by 0.5% to 0.980, which shows that the process of dynamic clustering and adaptive resource allocation during flow table overloading attacks is vital. Furthermore, the performance drops greatly when the DDST-IV module is removed, with an accuracy of 0.973 (−1.22%) as it plays an important role in feature extraction. In particular, the absence of the DCLSTM classifier leads to the worst performance with an accuracy of 0.959 (−2.64%). In addition, the deletion of the ICWO optimization technique slightly reduces the performance accuracy to 0.969 (−1.62%), showing that the presented approach really enhances the parameters’ tuning process, the stability of convergence, and detection robustness; the increase in the detection time is still reasonable for SDN architectures. Altogether, the findings from the ablation research show that each module really helps to enhance the intrusion 5G-SDN detection capabilities.

**Table 17:** Ablation analysis of the proposed model by removing the individual component.

Models	Accuracy (±0.002)	Precision (±0.003)	Recall (±0.002)	F1-score (±0.003)	Detection time (s)
Full proposed model	0.985	0.984	0.986	0.985	1.74
Without APCHC	0.980 (−0.5%)	0.978 (−0.61%)	0.980 (−0.6%)	0.979 (−0.61%)	1. 65
Without DDST-IV	0.973 (−1.22%)	0.953 (−3.15%)	0.961(−2.54%)	0.958 (−2.74%)	1.40
Without DCLSTM	0.959 (−2.64%)	0.934 (−5.08%)	0.923 (−6.3%)	0.928 (−5.79%)	1.38
Without ICWO	0.969 (−1.62%)	0.962 (−2.24%)	0.971 (−1.52%)	0.967 (−1.83%)	1.56

Large-scale multiclass IDS must be considered in the context of the performance losses presented in spite of their apparent quantitative modesty. Small percentage gains in performance will mean considerable development of the techniques of feature representation, optimization and class separation capability as the system approaches the point of saturation. It is important to note that the experiments were conducted using 23,670 testing samples of SDN intrusion dataset. In such conditions, the performance change of 1–3% means a considerable amount of better classified network flows. The loss of 1% of accuracy on the testing data set, for example, would mean about 200 more misclassified examples because of incorrectly identified network traffic or undetected attacks.

Table 18 presents the comparison between the performance of the proposed ICWO optimization algorithm and the performance of other available optimizers, such as Adam, Genetic Algorithm (GA) and Particle Swarm Optimization (PSO). Experimental analysis shows that the ICWO outperforms other algorithms in terms of optimization, with respect to improved classification accuracy, convergence, and training time. While the Adam optimizer gives competitive results due to adaptive gradient learning, the proposed ICWO optimizer yields slightly better performance in terms of optimization stability and better exploitation-exploration trade-off for parameter tuning. The performance of the GA-based optimizer is comparatively poor due to slow convergence and limited ability to solve high-dimensional nonlinear optimization problems. The same holds true for the PSO algorithm, whose performance is better than that of GA but faces an issue of premature convergence. The incorporation of ICWO is meant to act as an auxiliary approach that would help solve the problems posed by the proposed architecture in particular, instead of serving as a universal replacement for classical optimizers. The effectiveness of ICWO is based on the ability of this approach to cope with the complexity of the interaction between the time modelling, encoding, and clustering stages.

**Table 18:** Performance comparison of ICWO with the existing optimization algorithms.

Optimizer	Accuracy	Convergence epoch	Training time (s)
Adam (Standard DL Optimizer)	0.961	38	454
GA (Genetic Algorithm)	0.968	32	521
PSO (Particle Swarm Optimization)	0.972	35	537
ICWO (proposed)	0.985	25	534

With the application of chaotic dynamics to enhance diversity in the initial stages of the search process, the ICWO technique has improved the convergence characteristics of the original Walrus Optimization technique. Early convergence to local optima is avoided, and this technique makes the algorithm better at exploring the search space. The ICWO technique has improved the local search capability and the speed of convergence towards optimal solutions as the algorithm converges on potential regions during the process of the optimization. By empirically evaluating convergence for ICWO compared to Walrus Optimization Algorithm and other opponent optimizers, it can be noted that ICWO has a better fitness gain at the initial stage of the process, a faster convergence rate, and fewer oscillations. Moreover, the convergence plots show that the convergence is relatively more stable, and high-quality solutions are consistently achieved through several trials. Considering all the factors, the convergence behavior of ICWO has shown that ICWO has made a successful trade-off between search intensification and diversification to achieve efficient optimization and high-quality solutions. Therefore, the ICWO-based optimisation has improved convergence efficiency, network training stabilization and enhanced classification of attacks in the dynamic 5G-SDN traffic environment.

## 6 Discussion and limitations

### 6.1 Discussion of computational efficiency, scalability, and attack detection effectiveness

Experimental results indicate that the proposed framework is effective in attack detection and classification in the 5G SDN environment. In particular, the combination of the Affinity Propagated Hierarchical Clustering method, Deep Dual-Stream InfoVarCoder architecture and Dense Convolutional LSTM allows the system to have better feature representation and temporal learning abilities. Properly grouping network traffic patterns, the clustering process reduces redundancy and increases the discrimination ability of the DL model. Through processing traffic patterns in parallel streams, the Deep Dual-Stream InfoVarCoder architecture provides the possibility for the framework to simultaneously detect temporal dependencies and spatial correlations in the traffic data. It becomes possible due to the dual stream approach that significantly improves the model’s capability of distinguishing normal traffic from complex attacks, in particular in the 5G SDN environments that are characterized by high dynamics. Thus, the use of Dense Conv-LSTM allows increasing the resiliency and reliability of the proposed intrusion detection system through precise identification of different attacks. Moreover, the ICWO algorithm plays an important role in improving convergence in the training process and parameter optimization. This optimization algorithm helps to avoid premature convergence and increase global search abilities by introducing chaotic mapping procedures.

Based on the experimental analysis, the proposed design surpasses many DL approaches that have been reported in literature, demonstrating impressive performance with an accuracy of 0.985 ± 0.002 and a lower detection time of 1.74 s. It confirms the efficiency of the hybrid framework in detecting both known and unknown attacks in SDN-based 5G systems. Moreover, the design demonstrates a high capability in reducing false positives, which is a very important element for the development of a real-time security network. The combination of multiple learning and optimization approaches leads to the computational complexity of the proposed model. While comparing with existing detection methods, the clustering phase, two-stream feature extraction, and Dense Conv-LSTM temporal modelling require computing resources. But these components help learn representations in a more efficient manner and improve the capability of the model in recognizing various types of attack patterns. Moreover, at the time of training, the optimization process helps in achieving effective parameter tuning. Hence, the proposed system can be implemented in complex network security systems as it provides a balance between computational cost and detection effectiveness. The reason behind choosing APCHC, DDSTIV, DCLSTM, and ICWO as the components of the proposed system is that although they increase the computational cost, the improvements in the detection accuracy and feature extraction are much more important.

The experiments were conducted in varying conditions for SDN traffic flows in the range of 300–400 flows for the purpose of testing the scalability of the proposed framework. Using small detection times between 1.70 and 1.74 s, the proposed technique kept up high detection accuracy at 0.984 and 0.987 consistently. Throughput was kept high at 2,400–2,900 samples in all the tests, indicating efficient management of heavy load in the network. These results justify the appropriateness of the proposed framework in real-time and large-scale 5G-SDN intrusion detection scenarios, where the framework manages to maintain detection accuracy, computational efficiency and throughput under increasing load of traffic. Therefore, an efficient and scalable framework for securing the future generation of SDN-based 5G networks is obtained from clustering-based pre-processing, deep dual stream representation learning, temporal modelling with Dense Conv-LSTM and meta-heuristic optimization techniques.

### 6.2 Limitations and assumptions

There are still some limitations despite the fact that the simulation environment was meticulously crafted to incorporate relevant characteristics of the 5G-SDN environment, such as traffic management through the SDN controller, dynamic traffic flows, varied attack levels, and realistic interaction between protocols. Some of the examples of issues that may cause some behaviors not to be accurately reproduced in an artificial simulation environment include user mobility behaviors, vendor-specific network configurations, deployment-specific constraints, dynamic attack behavior, and unexpected traffic irregularities. Moreover, real networks tend to experience unexpected configuration changes, traffic irregularities, and environmental issues that are difficult to accurately simulate in a constrained modelling environment. Future research will involve testing the developed model for more practical applicability and generalization capability, future work will concentrate on validating it using actual network traces gathered from operational 5G-SDN deployments, even though the simulation framework offers a realistic and repeatable testbed for performance evaluation.

Despite the excellent detection accuracy, scalability, and real-time detection capacity of the proposed architecture, there are still a few difficulties. While compared to conventional ML methods, the combination of hierarchical clustering, dual-stream feature extraction, Dense Conv-LSTM modelling, and ICWO-based optimization raises the overall architectural complexity. The framework maintains reasonable training and detection times, according to the computational complexity analysis, but when implemented in very large-scale SDN environments with constantly increasing vast traffic volumes in the future 5G network, the clustering and optimization stages may add more computational overhead. In the same way, resource-constrained edge installations might not have the moderate memory and processing resources needed for the deep temporal learning components.

## 7 Conclusion

This research presents an efficient attack prediction, detection and defense mechanism for 5G-SDN networks, leveraging Affinity Cohesive Clustering and Dual Info-Varcoder with Dense ICWO-CLSTM for improved classification and threat detection. The proposed algorithm provides dynamic allocation of network resources against flow table overload attacks, thereby guaranteeing the continuous operation of the security process. The use of CLSTM with the ICWO is able to capture the spatial and temporal relationships, providing an excellent performance with an accuracy rate of 0.985 ± 0.002, precision of 0.984 ± 0.003, recall of 0.986 ± 0.002 and F1 score of 0.985 ± 0.003. It has a low RMSE of 0.3944 and a minimal log loss of 0.411 and MAE of 0.2120, which confirms its high prediction capabilities. The model has a fast training speed of 498.04 s and SDN detection time of 1.74 s and is capable of processing 3,218 samples per second while utilizing only 3.3% CPU. Low discrepancy between training loss and testing loss reflects its capability to learn well across different attacks like BF, CC, DDoS, and DGJY. This research reveals that the proposed algorithm holds great promise in securing future 5G systems by providing effective threat mitigation in advance.

## 8 Future scope

In future, this architecture will be upgraded to process live data feeds in order to dynamically adjust its behavior to new types of SDN attacks within 5G-SDN networks. The architecture of the model will be improved to allow processing multi-modal data from various edge and IoT devices, thus making it possible to detect threats in interconnected smart systems. In the future, efforts will be made to include federated learning and decentralized intelligence to increase the security level while maintaining the privacy of data. It is important to concentrate on scaling the network and resource consumption of attack detection models in the future. The more promising direction would be to use lightweight DL to reduce power consumption. Large-scale deployment of 5G networks will also be considered under high traffic conditions for upcoming AI—driven security in ultra-dense SDN networks in the future.

## Data Availability

The raw data supporting the conclusions of this article will be made available by the authors, without undue reservation.
